# Robust Glycoproteomics Platform Reveals a Tetra‐Antennary Site‐Specific Glycan Capping with Sialyl‐Lewis Antigen for Early Detection of Gastric Cancer

**DOI:** 10.1002/advs.202306955

**Published:** 2023-12-12

**Authors:** Luyao Liu, Lei Liu, Yan Wang, Zheng Fang, Yangyang Bian, Wenyao Zhang, Zhongyu Wang, Xianchun Gao, Changrui Zhao, Miaomiao Tian, Xiaoyan Liu, Hongqiang Qin, Zhimou Guo, Xinmiao Liang, Mingming Dong, Yongzhan Nie, Mingliang Ye

**Affiliations:** ^1^ CAS Key Laboratory of Separation Science for Analytical Chemistry, Dalian Institute of Chemical Physics Chinese Academy of Sciences Dalian 116023 China; ^2^ University of Chinese Academy of Sciences Beijing 101408 China; ^3^ The College of Life Sciences Northwest University Xi'an 710127 China; ^4^ State Key Laboratory of Cancer Biology, Xijing Hospital of Digestive Diseases Fourth Military Medical University Xi'an 710068 China; ^5^ MOE Key Laboratory of Bio‐Intelligent Manufacturing, School of Bioengineering Dalian University of Technology Dalian 116024 China; ^6^ State Key Laboratory of Medical Proteomics Beijing 102206 China

**Keywords:** gastric cancer, intact glycopeptides, MS‐based glycoproteomics, serum biomarkers, site‐specific N‐glycans

## Abstract

The lack of efficient biomarkers for the early detection of gastric cancer (GC) contributes to its high mortality rate, so it is crucial to discover novel diagnostic targets for GC. Recent studies have implicated the potential of site‐specific glycans in cancer diagnosis, yet it is challenging to perform highly reproducible and sensitive glycoproteomics analysis on large cohorts of samples. Here, a highly robust N‐glycoproteomics (HRN) platform comprising an automated enrichment method, a stable microflow LC‐MS/MS system, and a sensitive glycopeptide‐spectra‐deciphering tool is developed for large‐scale quantitative N‐glycoproteome analysis. The HRN platform is applied to analyze serum N‐glycoproteomes of 278 subjects from three cohorts to investigate glycosylation changes of GC. It identifies over 20 000 unique site‐specific glycans from discovery and validation cohorts, and determines four site‐specific glycans as biomarker candidates. One candidate has branched tetra‐antennary structure capping with sialyl‐Lewis antigen, and it significantly outperforms serum CEA with AUC values > 0.89 compared against < 0.67 for diagnosing early‐stage GC. The four‐marker panel can provide improved diagnostic performances. Besides, discrimination powers of four candidates are also testified with a verification cohort using PRM strategy. This findings highlight the value of this strong tool in analyzing aberrant site‐specific glycans for cancer detection.

## Graphical abstract

Aberrant glycosylation is recognized as a hallmark of cancers; however, no site‐specific glycan biomarker is available for clinical use. In this study, a highly robust N‐glycoproteomics (HRN) platform with remarkable stability is developed for biomarker discovery. A branched site‐specific glycan capping with sialyl‐Lewis antigen has been found to be a promising biomarker for the early detection of gastric cancer.

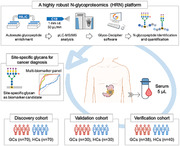

## 1 Introduction

Gastric cancer (GC) is one of the top five leading causes of cancer death worldwide, primarily due to late diagnosis, rapid metastasis, and less efficient therapeutics.^[^ ^1^, ^2^ ^]^ The diagnosis of GC mainly relies on endoscopy and biopsy, which inflicts pain and suffering.^[^ ^3^ ^]^ Serum‐based biomarkers of GC,^[^ ^2^ ^]^ including carcinoembryonic antigen (CEA), carbohydrate antigen 125 (CA) 125, CA72‐4, and CA19‐9, are either glycoproteins or glycan antigens, providing noninvasive diagnostics in the current clinical utility; however, they have poor diagnostic performances for early‐stage GC.^[^ ^4^ ^]^ Protein glycosylation is the most abundant and complex post‐translational process in eukaryotic cell proteins. In contrast to nucleic acid and proteins, the glycan biosynthesis of glycosylation is highly sensitive to the physiological state as it occurs by a complex network of metabolic and enzymatic reactions without a template.^[^ ^5^ ^]^ Aberrant glycosylation is well associated with diseases and has great potential for tumor reporting.^[^ ^6^, ^7^ ^]^ For instance, the core‐fucosylated fraction of α‐fetoprotein (AFP‐L3), an FDA‐approved biomarker, has been found to be elevated in the initial tumor stages of hepatocellular carcinoma (HCC), thus improving early detection compared to total AFP.^[^ ^8^, ^9^ ^]^ Protein fibulin‐3 has been found to increase its interaction with epidermal growth factor receptors (EGFR) when modified with CA19‐9, a tetra‐saccharide sialyl‐Lewis^a^ antigen, resulting in severe pancreatitis in mice models.^[^ ^10^ ^]^ Thus, it is essential to investigate specific glycosylation changes for insight into disease mechanisms and disease‐related biomarker discovery.

There has been a growing interest in mass spectrometry (MS)‐based glycoproteomics as a powerful tool to investigate site‐specific glycan changes in diseases.^[^ ^11^ ^]^ Site‐specific glycans contain three levels of information, i.e., the glycosites, the attached glycans, and the carrier proteins, which could be obtained by analyzing intact glycopeptides in MS‐based glycoproteomics. Recent advances in sample preparations, data acquisition methods, and spectra deciphering software have greatly facilitated highly sensitive profiling of site‐specific glycosylation in cancer research.^[^ ^12^, ^13^, ^14^, ^15^, ^16^, ^17^ ^]^ For instance, Cao et al. detected upregulated core‐fucosylation at the N‐glycosite 603 of EGFR in HCC via a sequential treatment of intact glycopeptides with enzymes (STAGE) strategy.^[^ ^12^ ^]^ Li et al. detected the increase of LacdiNAc‐containing N‐glycans on 10 glycosites of 8 glycoproteins as a feature of intrahepatic cholangiocarcinoma using isotopic labeling‐based quantitative *N*‐glycoproteomic analysis.^[^ ^13^ ^]^ These studies highlighted the potential of site‐specific glycans in tumor diagnosis using novel glycoproteomics approaches.

The discovery of diagnostic and prognostic markers generally require the analysis of a large number of clinical samples to improve accuracy and efficiency.^[^ ^18^ ^]^ Despite the development of novel MS‐based technologies, most clinical studies for detecting intact glycopeptides typically have small sample sizes (less than 100 patients). Moreover, intact glycopeptides have high microheterogeneity, low abundance, and low ionization efficiency compared to tryptic peptides, which makes site‐specific glycoproteomics analysis still a significant challenge. Besides, large‐scale analysis of intact glycopeptides has always been limited by the tedious process of manual enrichment, the instability of liquid chromatography‐tandem mass spectrometry (LC‐MS/MS) system in a long‐running process, and the poor data deconvolution of spectra deciphering. Therefore, it is urgent to develop a robust and sensitive site‐specific glycoproteomics workflow for the expansion of clinical research. In addition, it is crucial to identify site‐specific glycan biomarkers from human serum samples. Glycan profiling, glycopeptide profiling, and intact protein profiling of purified serum haptoglobin (HPT) of GC have been investigated.^[^ ^19^ ^]^ These studies focused on purified proteins are unable to discover biomarkers other than HPT. Unfortunately, the abnormal site‐specific glycosylation of GC at proteomics scale has hardly been studied.

Herein, we present a highly robust N‐glycoproteomics (HRN) platform for analyzing site‐specific glycans of a large cohort of samples for biomarker discovery study. In the HRN platform, intact N‐glycopeptides of biological samples were enriched through an automated hydrophilic interaction chromatography (HILIC) method and then analyzed by a microflow LC‐MS/MS system, followed by quantitative characterization using Glyco‐Decipher software. It was applied to analyze 200 clinical specimens of discovery and validation cohorts by shotgun glycoproteomics (or discovery glycoproteomics), and another 78 specimens of an independent verification cohort by targeted glycoproteomics. The HRN platform exhibited high sensitivity, reproducibility and stability throughout the clinical N‐glycoproteome analysis. A total of 21711 unique site‐specific glycans from 971 glycoproteins were detected from discovery and validation cohorts, providing in‐depth coverage of human serum glycoproteome. By employing differential abundance analysis and machine learning, four site‐specific glycans (AACT‐N^106^‐H7N6S4F1, A1AT‐N^271^‐H6N4S2, IC1‐N^352^‐H4N3S1, and ITIH3‐N^91^‐H5N4S2) were determined as promising biomarker candidates in detecting GC. Notably, AACT‐N^106^‐H7N6S4F1 has a special branched tetra‐antennary glycan structure capping with sialyl‐Lewis antigen, and it significantly outperformed serum CEA with the area under the curve (AUC) values > 0.85 compared against < 0.55 for the detection of GCs in three cohorts. The discrimination power of four candidates were further evaluated using parallel reaction monitoring (PRM)‐based strategy. And a four‐marker panel in particular showed superior diagnostic performances, with AUC values of 0.956 for GC, and 0.955 for early‐stage (stage I) GC. Our study demonstrated that the robust and sensitive platform is a powerful tool in analyzing large‐scale N‐glycoproteome profiling. It successfully insights the N‐glycosylation changes in cancer and provides potential glycosylated biomarkers for early detection of GC.

## 2 Results

### 2.1 A Robust Platform for Site‐Specific N‐Glycoproteomic Profiling

**Figure 1:**
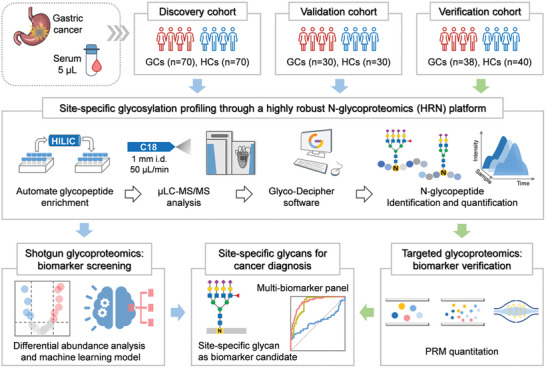
Workflow of serum site‐specific glycan biomarker discovery by a highly robust N‐glycoproteomics (HRN) platform. In the HRN platform, serum N‐glycopeptides were enriched by an automated HPLC‐HILIC method, detected by a stable microflow LC‐MS/MS system, and identified with high‐sensitive Glyco‐Decipher software. Site‐specific glycan biomarker candidates were determined from discovery and validation cohorts by shotgun glycoproteomics analysis and then confirmed in a verification cohort using targeted strategy.

**Figure 2:**
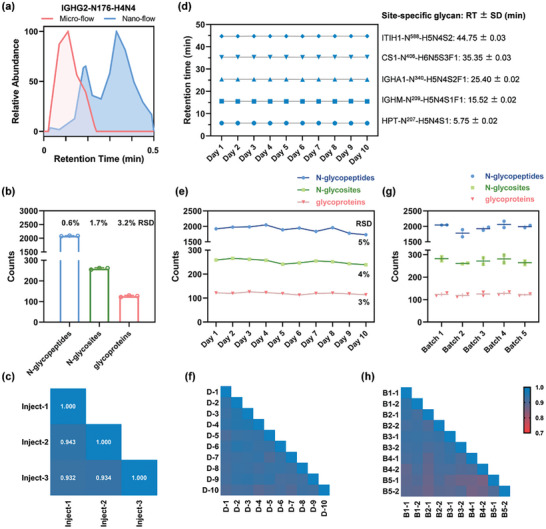
Performance evaluation of the HRN platform using quality control samples (QCs). a) Extracted ion chromatograms of the N‐glycopeptide, EEQFNSTFR with H4N4 (corresponding to IGHG2‐N176‐H4N4), analyzed by microflow and nanoflow LC‐MS/MS. b,e,g) The number of N‐glycopeptides, glycosites, and glycoproteins identified from triplicate injections b), ten MS‐QCs with one MS‐QC per day for ten days e), and ten batch‐QCs with two batch‐QCs per batch for a total of five batches f). c,f,h) Pearson correlation coefficients of quantified N‐glycopeptides of triplicate injections c), ten MS‐QCs f), and ten batch‐QCs h). d) Retention time of five N‐glycopeptides derived from MS‐QCs in ten days. SD, standard deviation.

We developed a highly robust N‐glycoproteomics (HRN) platform to quantitatively characterize site‐specific N‐glycans of a large cohort of samples in clinical trials (**Figure** **1**). The HRN platform adopted an automated strategy using durable HILIC columns for glycopeptide enrichment, a rapid and stable microflow LC‐MS/MS system for glycopeptide analysis, and the “pattern‐recognition” strategy for sensitive glycopeptide spectra deciphering. Nanoflow LC‐MS/MS is routinely used in proteomic studies, yet it suffers the issues of low reproducibility and stability.^[^ ^20^, ^21^ ^]^ Therefore, in order to facilitate extensive data acquisitions, the microflow LC‐MS/MS system was adopted as a novel approach for the detection of intact N‐glycopeptides. To test the microflow LC separation scheme, we loaded N‐glycopeptides of pooled human plasma onto a commercial C18 column of 1 mm inner diameter and eluted them with a flow rate of 50 µL min^−1^. For comparison, in the nanoflow scheme, the same glycopeptides were separated on a C18 column of 150 µm inner diameter with a flow rate of 600 nL min^−1^. The above two LC systems coupled to the same MS instrument, respectively. It can be seen from the base peak chromatograms that the microflow system started glycopeptide separation directly after sample injection, while the nanoflow system typically spent excess time for sample loading (Figure S1a, Supporting Information). In 60 min gradient time, the microflow LC resulted in narrower peak widths than the nanoflow LC (**Figure** **2**a; Figure S1b, Supporting Information). These findings suggested that the microflow system compared favorably against the nanoflow system by longer effective separation time, higher efficiency, and better peak shapes, which facilitated the accurate determination of peak area for quantification. As expected, the microflow system achieved better glycopeptide quantification reproducibility in triplicate with a median coefficient of variation (CV) of 6.6%, compared to the nanoflow of 13.4% (Figure S1c, Supporting Information). Our results were also consistent with previous studies that larger column diameters can improve the separation efficiency with higher peak capacity in general proteomics analysis.^[^ ^20^, ^22^ ^]^

Besides, we identified a comparable number of N‐glycopeptides (1558 vs. 1368) from 1 µL of the starting plasma using the nanoflow system and from 5 µL of the same sample using the microflow system, respectively (Figure S1d, Supporting Information). Although the required volume was different, it is easy to acquire a microliter scale of blood samples in the clinic. Loading amount of glycopeptides for microflow LC‐MS/MS analysis is well compatible with that for the front‐end glycopeptide enrichment. In the enrichment system, digested peptides from 5 µL of blood samples are automatically loaded and separated via an optimized 20 min gradient on a high‐performance liquid chromatography (HPLC)‐HILIC column, where non‐glycopeptides are removed in advance and glycopeptides are subsequently eluted and collected, followed by system re‐equilibrium. This automated method has been shown to have high enrichment specificity and outstanding reproducibility, and is an effective choice for glycopeptide preparation of a large number of biological samples.^[^ ^23^ ^]^ Moreover, Glyco‐Decipher software with spectrum expansion strategy could achieve higher sensitivity for glycopeptide identification compared to other glycoproteomics software (Figure S1d–h, Supporting Information). Taken together, the HRN platform ensures high reproducibility for glycopeptide enrichment by the automated method, good separation stability for glycopeptide detection by the microflow LC, and high sensitivity for glycopeptide identification by Glyco‐Decipher, which provides the potential for large‐scale N‐glycoproteome characterization.

### 2.2 Quality Assessment of the HRN Platform in a Large‐Scale Clinical Study

Repeatability is critical in biomarker candidate screening, where a large sample size needs to be measured over an extended period of time. We applied the HRN platform to analyze serum N‐glycoproteomes of 200 subjects participating in a clinical study, where quality control samples (QCs) were utilized for methodology assessment. Data‐dependent acquisitions (DDA) through microflow LC‐MS/MS system of 200 serum N‐glycopeptide samples spanned over ten days, with one MS‐QC per day to evaluate the inter‐day (different days) system stability. Beforehand, MS‐QCs were analyzed for three times to examine the intra‐day (within day) stability. MS‐QCs were aliquots of pooled serum N‐glycopeptide samples. The HRN platform performed well in terms of N‐glycoproteome identifications in triplicate (Figure 2b), with low relative standard deviation (RSD) of N‐glycopeptides (0.6%), N‐glycosites (1.7%), and glycoproteins (3.2%). And intra‐day glycopeptide quantification exhibited a high average Pearson correlation coefficient of 0.936 (Figure 2c). After long‐term running and large‐sample acquisitions, base peak chromatograms of ten MS‐QCs were virtually constant in ten days, indicating good stability of the chromatographic system and the electrospray (Figure S2, Supporting Information). The retention time for five highly abundant glycopeptides were nearly unchanged across ten days, with median standard deviation (SD) of 0.02 min, revealing high separation stability (Figure 2d). The HRN platform consistently identified around 1900 intact N‐glycopeptides, 250 N‐glycosites, and 120 glycoproteins all with RSDs below 5% across ten MS‐QCs (Figure 2e). And the inter‐day glycopeptide quantification presented an average Pearson correlation coefficient of 0.913 (Figure 2f). The results of serum N‐glycoproteome characterizations over ten days are comparable to that of three consecutive injections, demonstrating that data acquisition of the HRN platform maintained a low variability across large sample series.

To assess reproducibility of the entire workflow of HRN platform including sample preparation, we tested variabilities of batch‐QCs. Batch‐QCs were aliquots of pooled human serum. 140 subjects from the clinical study were randomly divided into five batches before glycoproteomics processes. Each batch contained 28 subjects with both patients and healthy individuals, and two batch‐QCs. Protein digestion and glycopeptide enrichment for each batch were conducted in different periods, while microflow LC‐MS/MS analysis for all samples was performed continuously over eight days. RSDs of the number of N‐glycopeptides, glycosites, and glycoproteins identified from ten intra/inter‐batch QCs were below 7% (Figure 2g). The average Pearson correlation coefficient for quantified N‐glycopeptides of batch‐QCs was 0.909 (Figure 2h). Besides, enrichment specificities of MS‐QCs and batch‐QCs which were interspersed in over 220 samples averaged 90%, ranging from 88.1% to 91.7%, revealing stable glycopeptide enrichment of the HRN platform (Figure S3, Supporting Information). In summary, the good reproducibility of the entire HRN platform lays a good foundation for its clinical application.

### 2.3 Overview of Study Populations for Serum N‐Glycoproteome Profiling

Gastric cancer (GC) is a common cause of cancer‐related death and studies have found that GC patients have altered serum 𝑁‐linked glycosylation compared to healthy state.^[^ ^24^ ^]^ To investigate site‐specific N‐glycosylation changes and to identify potential biomarkers of GC, we analyzed serum samples from 278 participants of three cohorts (**Table** **1**): 1) a discovery cohort contained 70 GC patients (GCs) and 70 healthy controls (HCs); 2) a validation cohort contained another 30 GCs and 30 HCs; and 3) an independent verification cohort consisted of 38 GCs and 40 HCs. In three cohorts, HCs were age‐ and gender‐matched to GCs (Figure S4, Supporting Information). Serum N‐glycoproteome characterization of each sample was implemented by the HRN platform. For biomarker screening, we acquired serum N‐glycoproteome profiles of the discovery and validation cohorts using shotgun glycoproteomics. And for biomarker verification, we processed the verification cohort using targeted glycoproteomics.

**Table 1:** Clinical characteristics of the discovery, validation and verification cohorts.

Characteristic	Discovery cohort		Validation cohort		Verification cohort	
	GCs (n = 70)	HCs (n = 70)	GCs (n = 30)	HCs (n = 30)	GCs (n = 38)	HCs (n = 40)
Gender (male/female)	44/26	40/30	18/12	17/13	25/13	25/15
Age (mean ± SD)	57.9 ± 14.2	58.4 ± 12.8	56.7 ± 16.0	56.7 ± 12.5	54.4 ± 11.0	45.3 ± 13.4
Serum CEA, ng/mL (mean ± SD)	17.1 ± 56.5	2.8 ± 1.8	25.3 ± 67.4	2.9 ± 1.6	31.0 ± 66.7	2.0 ± 1.2
TNM stage I/II/III	24/23/23	NA	10/10/10	NA	20/8/10	NA

### 2.4 Aberrant Site‐Specific N‐Glycosylation in GC and Biomarker Candidate Screening

**Figure 3:**
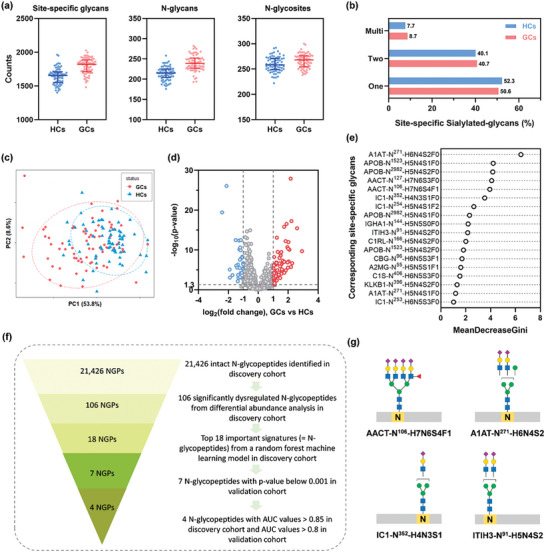
Serum N‐glycoproteome analysis and site‐specific glycan biomarker screening for GC. a) The number of site‐specific glycans, N‐glycans, and N‐glycosites identified in discovery cohort. b) Distribution of sialylated site‐specific glycans with one, two, or multi sialic acids. c) Principal‐component analysis (PCA) at quantified N‐glycopeptide level. d) Volcano plot comparing glycopeptides of GCs versus HCs. e) Top 18 glycopeptides ranked by the mean decrease of Gini index in random forest machine learning analysis. f) Entire process for biomarker candidate screening. g) Tentative site‐specific glycan structures of AACT‐N^106^‐ H7N6S4F1, A1AT‐N^271^‐H6N4S2, IC1‐N^352^‐H4N3S1, and ITIH3‐N^91^‐ H5N4S2.

In order to screen discriminating biomarkers, we implemented the “rectangular” biomarker discovery strategy in which biomarker candidates showed statistically significant differences between GCs and HCs in two cohorts using the same proteomics technology.^[^ ^25^ ^]^ Therefore, we analyzed the discovery and validation cohorts using the shotgun glycoproteomic approach in the HRN platform. From the discovery cohort, we identified 21 426 intact N‐glycopeptides and 20 001 site‐specific N‐glycans in total, corresponding to 1299 N‐glycans and 1261 N‐glycosites in 903 glycoproteins. Comparing N‐glycoproteome identifications, the median number of site‐specific glycans and N‐linked glycans identified from GCs were higher than HCs, while the median number of glycosites was comparable between the two groups (**Figure** **3**a). Thus, GC serum showed a significant degree of glycosylation alterations, mainly from more diverse glycan changes.^[^ ^24^ ^]^ N‐glycoproteome characterization via the HRN platform provides a great opportunity to probe glycosylation microheterogeneity by localizing glycan modifications to specific glycosites. We observed that over 50% of glycosites contained more than one N‐linked glycan in both GCs and HCs (Figure S5a, Supporting Information). And GCs had more glycoproteins with over ten N‐glycans at one glycosites than HCs (101 vs. 91, Figure S5b, Supporting Information), demonstrating more abnormal glycosyltransferase activity resulting in higher glycosylation microheterogeneity in the gastric tumor.

We then grouped site‐specific glycans according to N‐linked glycoforms: high mannose, sialylated glycans, and fucosylated glycans. The overall proportions of different types of site‐specific glycans in GCs were comparable to that of HCs (Figure S5c and d, Supporting Information). More multiple sialylations of site‐specific glycans were observed in the serum of GC since the ratio of di‐ and multi‐sialylated glycans was higher in GCs (49.4%) than HCs (47.7%) (Figure 3b). These results were in accordance with other studies that gastric mucosa expressed mostly neutral fucosylated glycans at healthy state but increased sialylation in gastric diseases.^[^ ^26^ ^]^

From the quantitative glycoproteomes, Pearson correlation coefficients of quantified N‐glycopeptides across 140 individuals of the discovery cohort were much lower than QCs (Figure S6, Supporting Information), revealing high personal differences. Note that Pearson correlation coefficients across 70 GCs were lower overall than those across 70 HCs, indicating a much higher glycosylation variability in GC. Unsupervised principal component analysis (PCA) for quantified N‐glycopeptides showed a degree of clustering between patients and controls (Figure 3c), where GCs were more dispersed in PCA space than HCs, highlighting a higher degree of glycosylation heterogeneity in GC. Further, differential abundance analysis revealed that 106 glycopeptides were significantly dysregulated (p‐value < 0.01, absolute log2 fold change > 1, Table S5, Supporting Information) in GCs versus HCs (Figure 3d). These dysregulated glycopeptides could form two distinct clusters in GCs and HCs (Figure S7, Supporting Information). Moreover, a total of 28 unique glycan compositions were derived from 106 dysregulated glycopeptides. Comparing glycopeptides that linked with the same glycans, more multi‐sialylated and multi‐fucosylated glycopeptides showed up‐regulation in GC (Figure S8a, Supporting Information). Also, up‐regulated glycopeptides modified with both fucosylation and sialylation (55%) showed higher proportion compared to down‐regulated (13%) and unchanged (27%) glycopeptides (Figure S8b, Supporting Information). Therefore, probing site‐specific glycans would be more informative for studying GC.

Random forest is a widely‐used machine learning algorithm that allows the efficient classification of large‐scale dataset with large number of features. More importantly, it enables the determination of the importance of each feature with model training. Hence, we chose random forest to screen biomarkers from the dysregulated intact glycopeptides that contributed to disease diagnosis. We built a random forest machine learning model based on the 106 significantly dysregulated N‐glycopeptide dataset for discriminating GC, leading to the prioritization of 18 important signatures (Figure 3e, Table S1, Supporting Information). Each important signature here represents one intact N‐glycopeptide. Further investigation showed that all important signatures were significantly altered between the GCs and HCs in the discovery cohort, with p‐values below 0.001 (Figure S9, Supporting Information). A validation cohort was further utilized to assess the biomarker candidates, of which all glycoproteomic experiments were performed separately and independently from the discovery cohort. In total, we identified 12 511 intact N‐glycopeptides and 11626 site‐specific glycans in 926 glycoproteins. The number of site‐specific glycans and N‐glycans were much higher in GCs than HCs, while N‐glycosites showed no apparent difference between groups, and these features were similar as that of discovery cohort (Figure S10, Supporting Information). Differential abundance analysis of the 18 signatures between GCs and HCs in the validation cohort determined seven ones with p‐value below 0.001 (Figure S11, Supporting Information). Therefore, the corresponding seven site‐specific glycans (A1AT‐N^271^‐H6N4S2, AACT‐N^106^‐H7N6S4F1, IC1‐N^352^‐H4N3S1, ITIH3‐N^91^‐H5N4S2, A2MG‐N^55^‐H5N5S1F1, KLKB1‐N^396^‐H5N4S2, and A1AT‐N^271^‐H5N4S1) were retained for further evaluation. Then, we determined the area under the curve (AUC) of the receiver operating characteristic (ROC) curve for the 18 important signatures in both cohorts (Table S2, Supporting Information). Most of these signatures yielded favorable performance with AUC values greater than 0.8 in the discovery cohort. On the basis of the seven signatures that were significantly altered in both cohorts, four signatures yielded outstanding diagnostic performance in the discovery cohort (AUCs > 0.85) as well as in the validation cohort (AUCs > 0.8). Hence, their corresponding four site‐specific glycans (A1AT‐N^271^‐H6N4S2, AACT‐N^106^‐H7N6S4F1, IC1‐N^352^‐H4N3S1, and ITIH3‐N^91^‐H5N4S2) were considered as biomarker candidates and retained for further analysis. The complete process for biomarker candidate screening was illustrated in Figure 3f.

### 2.5 Site‐Specific Glycan Biomarker Candidates in Detecting GC

**Figure 4:**
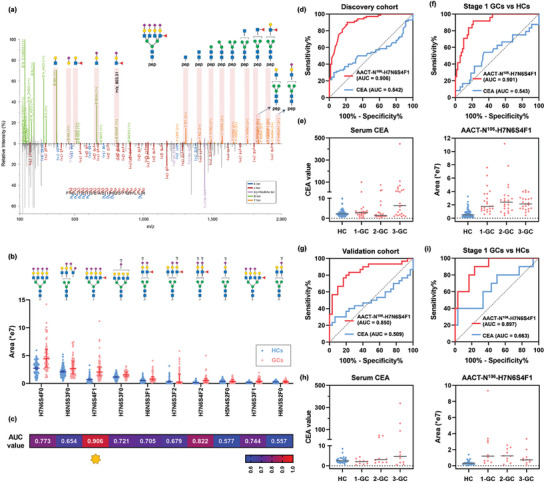
AACT‐N^106^‐H7N6S4F1 as a diagnostic signature of GC. a) Representative MS2 spectrum of glycopeptide “FNLTETSEAEIHQSFQHLLR linked with H7N6S4F1”, which corresponds to AACT‐N^106^‐H7N6S4F1. Paired spectrum with B/Y fragment ions (top) and b/y fragment ions (bottom). Expression distributions b) and AUC values for GC diagnosis c) of top 10 abundant glycans on AACT site 106 in discovery cohort. In discovery cohort, ROC curves for diagnosing GCs d) and early‐stage GCs f), and abundance distributions of serum CEA and AACT‐N^106^‐H7N6S4F1 e). In validation cohort, ROC curves for diagnosing GCs g) and early‐stage GCs i), and abundance distributions of serum CEA and AACT‐N^106^‐H7N6S4F1 h).

In addition to glycan compositions, specific glycan structures also play crucial roles in determining the biological property of their carrier proteins. Thus, characterizing aberrant glycan structures may provide additional information on the development and progression of cancer. Glyco‐Decipher software of the HRN platform could score and achieve rough determination of N‐glycan structures by matching diagnostic B/Y ions in experimental spectra against theoretical B/Y ions deduced from the fragmentation of glycan structures encoded in WURCS 2.057 format.^[^ ^27^ ^]^ Based on this, the probable glycan structures (Figure 3g) of four biomarker candidates were investigated based on MS2 spectra (Figure S12, Supporting Information). In brief, A1AT‐N^271^‐H6N4S2 and AACT‐N^106^‐H7N6S4F1 both had highly branched (tri‐ and tetra‐antennary) N‐glycan structures. AACT‐N^106^‐H7N6S4F1 also had a terminal sialyl‐Lewis antigen, and its typical MS2 spectrum contained a diagnostic ion at m/z 803.30 (H1N1S1F1), providing evidence for the terminal sialyl‐Lewis structure (**Figure** **4**a).

Increases in fucosylation, branching, and sialylation of N‐glycans are often observed in cancer, and especially N‐glycan branching structures are frequently upregulated in GC.^[^ ^28^ ^]^ Synthesis of N‐glycan branches in cells is catalyzed by several GlcNAc transferases (GnT‐III, GnT‐IVs, GnT‐V, GnT‐IX) to provide functionally diverse N‐glycoproteins, of which aberrant formation has currently been reported to be involved in tumor growth, invasion, and metastasis.^[^ ^28^ ^]^ Among them, GnT‐V and GnT‐IV produce β1,6‐ and β1,4‐branch on N‐glycans, respectively, leading to the synthesis of tri‐ and tetra‐antennary structures. In particular, GnT‐V expression was detected by immunohistochemistry in GC tissues and was found to be significantly associated with poor prognosis of GC patients, showing that increased β1,6‐branch N‐glycan levels of cell proteins contribute to metastases.^[^ ^29^ ^]^ GnT‐IV expression is dysregulated in several cancer cells or tissues (such as pancreatic cancer, and hepatocarcinoma), suggesting that β1,4‐branch N‐glycans promote invasion and metastasis.^[^ ^30^, ^31^, ^32^ ^]^ Besides, sialyl‐Lewis antigens containing two isomers (sialyl‐Lewis^a^ and sialyl‐Lewis^x^) are common terminal antigens that are overexpressed in many types of solid tumors and are correlated with metastasis and poor patient survival.^[^ ^33^ ^]^ For instance, CA19‐9, a biomarker for monitoring GC progression, has been determined to be a tetra‐saccharide antigen with a specific sialyl‐Lewis^a^ structure. In general, human healthy gastric mucosa expresses neutral glycans with terminal Lewi's antigens. However, H. pylori‐mediated inflammation induces overexpression of sialylated and sulfated glycans of Lewis antigens resulting in remodeling of the glycosylation profile of gastric cells. Further, malignant transformation of infection is accompanied with an increased expression of sialyl‐Lewis^a^ and sialyl‐Lewis^x^ antigens for cell invasion and metastasis.^[^ ^34^ ^]^ Therefore, we conducted dedicated analysis on AACT‐N^106^‐H7N6S4F1 since it has highly sialylated and tetra‐antennary branched N‐glycan structures capping with terminal sialyl‐Lewis antigen.

We wondered whether other site‐specific glycans on AACT site 106 had similar diagnostic power as AACT‐N^106^‐H7N6S4F1. A total of 159 unique N‐glycans were detected on AACT site 106 due to the high sensitivity of the HRN platform, indicating high heterogeneity of glycosylation on AACT. The top ten most abundant glycans on AACT site 106 were selected to test their diagnostic performance in the discovery cohort (Figure 4b). Several site‐specific N‐glycans indeed had expression differences between GCs and HCs. Nevertheless, H7N6S4F1 showed the best diagnostic performance (AUC, 0.906; 95% CI, 0.857‐0.953; specificity, 90.0%; sensitivity, 78.6%) compared to any other N‐glycans on AACT site 106 (Figure 4c). Our results highlighted the value of site‐specific glycans as novel biomarkers in diagnosing GC.

Serum CEA is a frequently used biomarker for GC, which is primarily measured by the immunometric format with a cutoff value of 5 ng mL^−1^.^[^ ^35^ ^]^ However, in the discovery cohort, the diagnostic performance of serum CEA (AUC, 0.542; 95% CI, 0.443‐0.641; specificity, 30.0%; sensitivity, 94.3%) was much lower than that of AACT‐N^106^‐H7N6S4F1 (Figure 4d). In detail, all healthy individuals and 55 patients had CEA values < 10 ng mL^−1^, while the remaining 15 patients had CEA values > 10 ng mL^−1^ (Figure 4e). As a slight elevation of the serum CEA reveals less clinical significance, we divided patients into two groups by a new CEA cutoff value of 10 ng mL^−1^ that was twice the normal value. AACT‐N^106^‐H7N6S4F1 yielded AUC values of 0.921 (95% CI, 0.842‐1.000) for the detection of CEA‐positive GCs and 0.901 (95% CI, 0.850‐0.953) for CEA‐negative GCs, respectively (Figure S13, Supporting Information). These results demonstrated that AACT‐N^106^‐H7N6S4F1 is a CEA‐independent biomarker candidate and can be useful in supplementing CEA for GC diagnosis.

Since most GC patients are always asymptomatic until progressing to advanced stages, it is essential to develop effective screening approaches for the early detection of GC. Aiming for diagnostic biomarker discovery, all patients in the discovery and validation cohorts were evenly distributed from stage I to III (no stage IV, Table 1). Serum CEA values showed an upward trend from stage I to III with tumor progression, meanwhile, the expressions of AACT‐N^106^‐H7N6S4F1 increased in all three stages of GCs compared to HCs in the discovery cohort (Figure 4e). As expected, AACT‐N^106^‐H7N6S4F1 yielded superior performance in diagnosing each stage of GCs (AUCs > 0.9) compared to serum CEA (AUCs < 0.7) (Figure S14a, Supporting Information). Encouragingly, AACT‐N^106^‐H7N6S4F1 performed much better than serum CEA in diagnosing early‐stage GC, with AUC values of 0.901 (95% CI, 0.837‐0.964) versus 0.543 (95% CI, 0.398‐0.689) and sensitivities of 87.5% versus 29.2% under the same specificity of 80% (Figure 4f). The discovery and validation cohorts presented similar results. AACT‐N^106^‐H7N6S4F1(AUC, 0.850; 95% CI, 0.794‐0.951; specificity, 76.7%; sensitivity, 83.3%) still outperformed serum CEA (AUC, 0.509; 95% CI, 0.356‐0.662; specificity, 20.0%; sensitivity, 96.7%) in diagnosing GCs of the validation cohort (Figure 4g). Expression levels of serum CEA and AACT‐N^106^‐H7N6S4F1 in three stages of GC exhibited a similar trend in both cohorts (Figure 4h). Notably, for early‐stage GC detection, the performance of AACT‐N106‐H7N6S4F1 (AUC, 0.897; 95% CI, 0.794‐0.999) was much better than serum CEA (AUC, 0.663; 95% CI, 0.450–0.876) (Figure 4i). Also, it had greater sensitivity than CEA (80.0% vs. 40.0%) under the same specificity of 80%. Besides, this site‐specific glycan had stable performances in diagnosing patients with stage II and III (Figure S14b, Supporting Information). Together, the above results confirmed that AACT‐N^106^‐H7N6S4F1 could serve as a valuable biomarker candidate in diagnosing GC.

**Figure 5:**
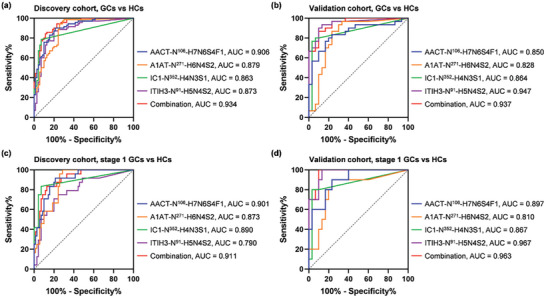
Site‐specific glycan biomarker panel in discovery and validation cohorts. ROC curves for diagnosing GCs a,b) and early‐stage GCs c,d) using each of the four site‐specific glycans and their combinations.

Besides AACT‐N^106^‐H7N6S4F1, three other site‐specific glycans (A1AT‐N^271^‐H6N4S2, IC1‐N^352^‐H4N3S1, and ITIH3‐N^91^‐H5N4S2) also demonstrated strong discrimination power for GC diagnosis, with AUC values > 0.8 in both cohorts. A combination of four site‐specific glycans as a biomarker panel presented a favorable diagnostic performance with an AUC value of 0.934 in the discovery cohort (**Figure** **5**a) as well as an AUC value of 0.937 in the validation cohort (Figure 5b). The biomarker panel with increased AUC values could well supplement AACT‐N^106^‐H7N6S4F1. Moreover, the panel offered superior performance for early‐stage GC detection with AUC values of 0.911 in the discovery cohort (Figure 5c) and 0.963 in the validation cohort (Figure 5d). Conclusively, AACT‐N^106^‐H7N6S4F1 can work either independently or combined with other site‐specific glycans, where the biomarker panel could gain improved discrimination power for GC diagnosis as well as provide improved diagnostic stability.

### 2.6 Biomarker Verification using Targeted Glycoproteomics in the HRN Platform

To verify the discrimination power of the aforementioned four site‐specific glycan candidates in detecting GC, we analyzed an independent verification cohort containing 38 GCs and 40 HCs (Table 1) using targeted glycoproteomics approach in the HRN platform. Serum N‐glycopeptide samples of the verification cohort were prepared using the automated enrichment method under the same conditions as two former cohorts, and then detected under the PRM acquisition mode in the microflow LC‐MS/MS system.

**Figure 6:**
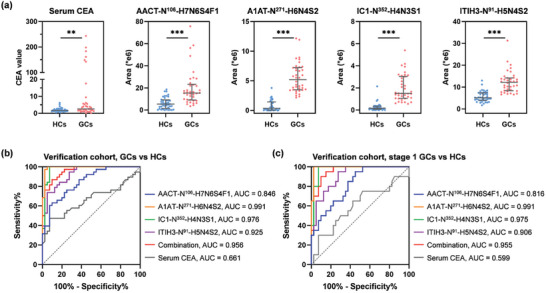
Biomarker verification using targeted glycoproteomics. a) Abundance distributions of serum CEA and four site‐specific glycans in verification cohort. Error bar, median with interquartile range. ROC curves for diagnosing GCs b) and early‐stage GCs c).

The expression levels and the diagnostic performances of serum CEA and four biomarker candidates were illustrated in **Figure** **6**. With only a small number of GC patients had serum CEA values > 10 ng mL^−1^, serum CEA yielded limited capacity in GC detection with AUC values of 0.661. On the contrary, the expression levels of four site‐specific glycans all significantly altered (p value < 0.001) in GCs relative to HCs with at least 2 times fold change (Figure 6a). Consequently, the four candidates demonstrated good performances in GC detection as an individual marker with AUC values > 0.85, which is substantially better than serum CEA. Notably, when combined together to form a four‐signature panel, an AUC value of 0.956 was achieved for GC detection (Figure 6b). For early‐stage GC detection, the biomarker panel outperformed serum CEA with an AUC value of 0.955 compared against 0.599 (Figure 6c). Conclusively, four site‐specific glycans and their combination offered strong discrimination power for GC in three different cohorts. Thus, aberrant site‐specific N‐glycans correlate well with diseases and have great potential as diagnostic biomarkers for early‐stage GC.

## 3 Discussion

Here, we developed a robust HRN platform and applied it to explore novel site‐specific glycan biomarkers for GC diagnosis. The HRN platform achieved intact glycopeptide enrichment on a HILIC column connected to an HPLC system. This automated mode for uninterrupted and continuous enrichment significantly reduces variation caused by manual operations, and is well‐suited for large‐scale experiments. Different clinical specimens tested in this work generated various numbers of oxonium ion‐containing spectra and the total MS2 spectra, but still yielded high enrichment specificities (90% on average), implying the robust performance of the enrichment strategy. The HRN platform also secured stable data acquisition through a microflow LC‐MS/MS system. Reliable and reproducible raw data collection is a prerequisite for large‐scale proteomics studies, especially for biomarker screening in cancer research. In most proteomics analysis, nanoflow LC‐MS/MS typically have high sensitivity in peptide detection but suffers the issue of low stability. To circumvent this problem, microflow LC‐MS/MS was implemented for large‐scale glycoproteomics analysis for the first time in this work. A systematic comparison between microflow and nanoflow LC‐MS/MS confirmed that the former could provide more stable and sharper chromatography peaks, thus benefiting the precise quantification of glycopeptides. Also, a larger internal diameter analytical column used in the microflow LC is more tolerant of contaminants, improving the system durability. Furthermore, a lower sensitivity of the microflow system could be partly solved by loading > 5 times the amount of peptides.^[^ ^21^, ^36^ ^]^ Accordingly, 5 µL of blood per sample were analyzed in this work, of which the glycopeptide identifications was comparable to 1 µL of blood using nanoflow system. It should be noted that such a microliter scale of human blood is readily available in routine clinical testing (5 µL serum equals 300–400 µg proteins).

Besides, the Glyco‐Decipher software also contributed to the high‐sensitivity glycopeptide identification of the HRN platform. The spectrum expansion strategy of Glyco‐Decipher improves the identification sensitivity of glycopeptide spectra with poor peptide fragmentation by exploiting the high similarity in peptide fragmentation patterns of the same backbone glycopeptides, which compensates for the limited glycoproteome depth of a single injection. Moreover, we used a stepped higher‐energy collisional dissociation (HCD) fragmentation strategy to generate a broad range of b/y and B/Y ions in the MS2 spectra for both peptide and glycan recognition. Each serum QC could steadily yield an average of 1900 intact N‐glycopeptides even when large numbers of specimens (>200) were analyzed over a long period of time. In contrast, most glycoproteomics studies reported far less than 1000 N‐glycopeptide identifications in the single‐shot analysis of serum/plasma samples when only intact N‐glycopeptides were analyzed.^[^ ^37^, ^38^, ^39^, ^40^ ^]^ Besides determining glycan compositions, Glyco‐Decipher could provide probable glycan structures by matching theoretical B/Y fragment ions with experimental B/Y ions in glycopeptide spectra, which helps to reveal the important glycan structure changes associated with tumor development.

Due to the lack of efficient biomarkers for early detection, GC patients are always diagnosed at advanced stages resulting in low survival rates.^[^ ^2^ ^]^ Several studies have analyzed glycans, glycopeptides, and intact protein of serum HPT in GC.^[^ ^19^ ^]^ But analysis of a single purified serum protein loses opportunity for the discovery of novel biomarkers from other glycoproteins. In addition, pooled serum of GC has been studied recently where five glycoproteins containing both significantly changes of site‐specific N‐ and O‐glycosylation were observed.^[^ ^41^ ^]^ But these important features discovered from pooled serum have not yet been validated by individual samples. Hence, we adopted the HRN platform to investigate serum N‐glycosylation changes in individual GC patients and to discover novel glycosylated biomarkers for GC diagnosis. In brief, we screened the glycosylated biomarker candidates from a discovery cohort (70 GCs and 70 HCs) and a validation cohort (30 GCs and 30 HCs) using shotgun strategy, and then verified the candidates in an independent verification cohort (38 GCs and 40 HCs) using targeted strategy. For biomarker screening, glycoproteomics profiling through DDA acquisition mode achieved deep coverage of serum glycoproteome, which could not only identify high microheterogeneity of glycosylation, but also distinguish diagnostic efficacy of different site‐specific glycans. The heterogeneity of glycans and site‐specific glycans was proven to be increased in GCs compared to HCs. In order to ensure the data quality, 1053 N‐glycopeptides had 70% valid values in at least one group of discovery cohort were reserved in data pre‐processing step. And upregulated glycopeptides of the patient group showed more missing values in HCs, which facilitated us to discover increased expression of potential biomarker that hardly expressed in healthy state (Figure S15, Supporting Information). According to comprehensive analysis for quantitative glycoproteomes of discovery and validation cohorts, four site‐specific glycans were determined as biomarker candidates with superior performance in distinguishing GCs. Encouragingly, a biomarker panel of four site‐specific glycans yielded AUC values over 0.9 for the detection of early‐stage GC.

For biomarker verification, candidates were tested in an independent cohort through PRM‐based acquisition mode. These four site‐specific glycan candidates offered AUC values over 0.84 individually for GC detection and AUC value of 0.956 when combined together as a panel. Notably, the biomarker panel demonstrated an AUC value of 0.955 for early‐stage (stage I) GC detection, which significantly outperformed serum CEA (AUC, 0.599). Note that the expression levels of the above biomarker candidates (or site‐specific glycans) were derived from the summed intensity of the precursor ions of their corresponding intact N‐glycopeptides using Glyco‐Decipher, namely MS1‐based quantification. Despite the fact that PRM assay generally employs fragment ions (MS2) for quantification, we explored the differences between MS1 quantification using Glyco‐Decipher and MS2 quantification using Skyline software (Figure S16, Supporting Information). Skyline recognizes Y and b/y ions with top‐N intensities to quantify the target glycopeptide (Table S4, Supporting Information). However, it is hard to distinguish different glycopeptides with similar glycans attached to the same peptide backbone. One typical example was two intact N‐glycopeptides, YLGNATAIFFLPDEGK linked with H6N4S2 and H6N4S1F2, respectively, of which the retention time were nearly the same and the difference of m/z values was less than 1 Da. Since most of Y ions of these two glycopeptides were identical, Skyline easily processed error identification of H6N4S1F2‐containing spectra as H6N4S2‐containing spectra (Figure S16d and e, Supporting Information). In contrast, Glyco‐Decipher identifies the parent ion isotope cluster patterns of the target glycopeptide during MS1 quantification, which ensures accurate detection. Therefore, we chose Glyco‐Decipher with higher resolution to identify and MS1‐based quantify targeted glycopeptides.

Our findings highlight that large‐scale N‐glycosylation profiling at site‐specific level offers exciting opportunities for disease biomarker study. Among the four biomarker candidates, we focused on AACT‐N^106^‐H7N6S4F1, a branched tetra‐antennary glycan structure capping with sialyl‐Lewis antigen. It outperformed serum CEA with AUC values > 0.82 compared against < 0.66 for the detection of early‐stage GCs in three cohorts. Besides, dysregulated expression of AACT and its glycan modification were also found in other types of diseases, including liver cancer, pancreatic cancer,^[^ ^42^ ^]^ lung cancer,^[^ ^43^, ^44^ ^]^ ovarian cancer,^[^ ^45^ ^]^ sepsis and septic episode.^[^ ^46^ ^]^ The mRNA expression level of AACT presented no obvious change for GC from the Gene Expression Profiling Interactive Analysis (GEPIA) database,^[^ ^47^ ^]^ indicating that AACT‐N^106^‐H7N6S4F1 alterations in GC may attribute to the differences in protein or glycosylation levels. As we inspected the top 10‐abundant glycans on AACT site 106, AACT‐N^106^‐H7N6S4F1 still had the best diagnostic performance with an AUC of 0.906 compared to other site‐specific glycans with AUCs < 0.83. This demonstrated that site‐specific glycosylation is a unique pattern for biomarker diagnosis.

The HRN platform currently requires 20 min for HILIC enrichment and 60 min for reversed‐phase liquid chromatography (RPLC)‐MS/MS analysis of each sample. It ensures highly‐sensitive identification of intact glycopeptides in complex samples for the biomarker discovery. However, for the biomarker validation phase with a large number of clinical samples, it is essential to further shorten the analysis time. We have attempted to reduce the RPLC‐MS/MS analysis time to 20 min. Unfortunately, glycopeptides sharing the same peptide backbones were virtually coeluted, which made the identification of glycopeptides with similar glycan mass impossible. Recently, we reported an integrated platform by using a HILIC column for the simultaneous enrichment and separation of N‐ and O‐linked intact glycopeptides from the same serum sample.^[^ ^41^ ^]^ It was demonstrated that HILIC enabled the separation of glycopeptides mainly based on their carried glycans, and therefore has the potential to tackle the glycopeptide co‐elution problem in RPLC‐MS/MS. Thus, it is worth exploring HILIC‐MS/MS system with short gradient to increase the throughput. Because the using of HILIC column, the glycopeptide enrichment could be integrated in the system, which may further improve the throughput and reproducibility. Meanwhile, advanced MS technologies could also be adopted to improve the throughput and performance. For example, Ion Mobility Spectrometry (IMS), with its ability to separate ions based on shape and collision cross section, can be applied to mitigate the co‐elution issues by distinguishing glycopeptides with the same peptide backbone but different glycans. It can be used in glycoproteomics analysis system for further improving the performance of site‐specific glycoform analysis.

In summary, we developed a highly robust and sensitive HRN platform for large‐scale quantitative N‐glycoproteome profiling. And we discovered and verified four site‐specific glycans as potential biomarker candidates for the detection of GC through three cohorts. Site‐specific glycan investigation at proteome level provides a novel sight for the abnormal synthesis of glycosylation in tumor cells as well as a better understanding for cancer‐associated. We expect that the HRN platform will have widespread use in basic and clinical glycoproteomic studies.

## 4 Experimental Section

### Chemical and Reagents

Urea, NH_4_HCO_3_, dithiothreitol (DTT), iodoacetamide (IAA), trifluoroacetic acid (TFA), formic acid (FA), and trypsin (bovine, TPCK‐treated) were purchased from Sigma (St. Louis, MO, USA). Acetonitrile (ACN, HPLC grade) was purchased from Merk (Darmstadt, Germany). Deionized water was generated by a Milli‐Q system (Millipore, Milford, MA).

### Clinical Sample Collection

The study protocol was approved by the Ethic Committee of the First Affiliated Hospital of the Fourth Military Medical University, Xi'an, China (Approved NO. of ethic committee: KY20192088‐F‐1). Written informed consent was obtained from all participants. The study design and conduct complied with all relevant regulations regarding the use of human study participants and was conducted in accordance with the criteria set by the Declaration of Helsinki. All healthy individuals were without indication of gastrointestinal tract cancer as identified through serum biomarker screening and CT scan. All clinical serum samples were collected in the bio‐sample bank of Xijing Hospital. GC patients and healthy controls in three cohorts (discovery, validation, and verification cohorts) were well‐matched for gender and age. The clinical characteristics are summarized in Table 1.

### Intact N‐Glycopeptide Enrichment in the HRN Platform

5 µL of individual human serum from GC patients, healthy controls, or QC samples were diluted ten times with 8 M urea/0.1 M NH_4_HCO_3_, and then denatured by reduction (with 20 mM DTT at 37 °C for 2 h) followed by alkylation (with 40 mM IAA at 25 °C for 40 min). After urea concentration of the mixture was reduced to below 2 M with 0.1 M NH_4_HCO_3_, protein digestion was carried out with trypsin at an enzyme‐to‐protein ratio of 1:50 (w/w) at 37 °C for 16 h. Tryptic peptides were subsequently desalted using Oasis HLB C18 cartridges (Waters) and then lyophilized. Glycopeptide enrichment and MS analysis of intra‐batch samples were performed randomly to avoid bias. Intact N‐glycopeptides were enriched by the automated N‐glycopeptide enrichment method, according to previously reported.^[^ ^23^ ^]^ In brief, the lyophilized tryptic peptides were redissolved in 0.1% TFA/80% ACN and injected automatically onto a HILIC column at a flow rate of 200 µL min^−1^. The glycopeptide fraction was collected after the non‐glycopeptide fraction was previously washed away. The following sample could be injected after system washing and re‐equilibration. The whole enrichment cycle for each sample needs 20 min.

### Microflow LC‐MS/MS Analysis in the HRN Platform

The enriched glycopeptides derived from 5 µL serum were resuspended in 0.1% FA and submitted for microflow LC‐MS/MS analysis, which was performed in an Ultimate 3000 LC system coupled online to an Orbitrap Exploris 480 mass spectrometer (Thermo Fisher Scientific, USA). Glycopeptides were separated on a commercially available Acclaim PepMap 100 C18 LC column (2 µm particle size, 1 mm inner diameter × 150 mm; catalog number 164 711, Thermo Fisher Scientific) at a flow rate of 50 µL min^−1^. The column temperature was maintained at 45 °C. The mobile phases comprised mobile phase A (0.1% FA) and mobile phase B (0.1% FA/80% ACN). The overall 60 min LC gradient was described as follows: held at 4% B for 0.5 min, from 4% to 9% B for 0.5 min, from 9% to 45% B for 51 min, from 45% to 95% B for 2 min, held on 95% B for 4 min to clean the system, and finally back to 4% B for 2 min to equilibrate the system.

For shotgun glycoproteomics, DDA mode was operated to switch between MS and MS/MS acquisition. Full scan MS spectra were collected from 350 to 1800 m/z at a resolution of 60 000, with a normalized AGC target of 300 and a maximum injection time (IT) of 25 ms. MS/MS scans were performed at a resolution of 30 000 using an isolation window of 2 m/z, with a normalized AGC target of 200 and a maximum IT of 100 ms. Glycopeptide fragmentation was performed by stepped HCD with normalized energy of 20%, 30% and 40%.

For targeted glycoproteomics, PRM mode was employed by an optimized MS setting. Full scan MS spectra were collected from 450 to 2000 m/z at a resolution of 60 000, with a normalized AGC target of 300 and a maximum IT of 100 ms. PRM MS/MS scans were performed at a resolution of 30 000 using an isolation window of 2 m/z, with a normalized AGC target of 500 and a maximum IT of 100 ms. The targeted precursor ions were derived from four biomarker candidates as listed in Table S3 (Supporting Information).

### Intact N‐Glycopeptide Characterization in the HRN Platform

For N‐glycopeptide identification, raw data were searched with Glyco‐Decipher software^[^ ^27^ ^]^ against the human UniProt database (20404 protein entries released in 2019_03). The spectra were searched using precursor and fragment ion tolerance of 10 and 20 ppm, respectively. The search was restricted to tryptic peptides allowing up to three missed cleavages. Cysteine carbamidomethylation (C +57.022 Da) was specified as a fixed modification. Methionine oxidation (M +15.995 Da) was set as a variable modification. False discovery rate (FDR) of glycopeptide spectrum match (GPSM) was restricted to less than 1%. The detailed identification algorithm of Glyco‐Decipher was provided in Supplementary Methods of Supporting Information.

For MS1‐based quantification, Glyco‐Decipher reported the relative abundance of each glycopeptide according to the precursor peak area derived from the MS1 elution profile of the DDA and PRM raw files. And the relative quantitation of site‐specific glycans was calculated by the sum of glycopeptides bearing the same glycan on the same glycosite with different peptide backbones. For MS2‐based quantification, Skyline software was employed for peak integration and quantification of the selected glycopeptides from the PRM raw files. Fragment ions with high intensity of each targeted glycopeptide were derived from DDA raw data. All peaks were manually corrected by comparing the identification results of Glyco‐Decipher to ensure the correct detection of parent and fragment ions. The targeted peptide sequences were inserted firstly, and possible glycan chains as variable modifications were added to the Asn residue of peptide. In the transition settings, ion types were set as b, y ions, the precursor charges were set as +3 to +6, the ion charges were set as +1 to +3, and special ions where a series of Y fragment ions (i.e., Y0, Y‐HexNAc(1), Y‐HexNAc(2), Y‐Hex(1)HexNAc(2), Y‐Hex(2)HexNAc(2), and Y‐Hex(3)HexNAc(2)) were added. The ion match tolerance was set as 10 ppm for parent ions and 20 ppm for transitions. Skyline reported the integral peak area of selected ions as the relative abundance of targeted glycopeptides for further glycopeptide quantification.

### Differential Abundance Analysis and Random Forest Model

Quantified glycopeptides with 70% valid values in at least one group in the discovery cohort were reserved for downstream statistical analysis. After data pre‐processing, 1053 intact N‐glycopeptides were retained. Missing values were then treated with zero‐imputation. The student's t‐test was performed for GC patients and healthy controls using Perseus (version 1.6.0.7) software.^[^ ^48^ ^]^ Differentially expressed glycopeptides were determined using the criteria of p‐value < 0.01 and absolute log2 fold change > 1. Significantly expressed glycopeptides were selected for building a random forest machine learning model. In the random forest analysis, 500 trees were built using the R package RandomForest (version 4.7‐1.1) with 10‐fold cross‐validation. Random forest employed the out‐of‐bag method to estimate for the error rate, where 357 trees had the lowest error rate. Therefore, in this step, the forest error rate was decreased to ensure a stronger classifier. And then the random forest was constructed with an optimal number of trees and the feature importance was estimated by Gini index from the random forest. Top‐N important signatures were selected according to the mean decrease Gini values for further investigation. All receiver‐operating characteristic (ROC) curves and area under the curve (AUC) calculations were performed using the R package pROC.^[^ ^49^ ^]^ The long names of monosaccharides were replaced with single‐letter codes (H, Hex; N, HexNAc; S, NeuAc; F, Fuc). The hierarchical clustering of differential abundance across patients and controls was presented as a heatmap generated by ClustVis.^[^ ^50^ ^]^ Other bat plots and box plots were generated using GraphPad Prism (version 8.0.2, for Windows, GraphPad Software, San Diego, California USA, www.graphpad.com)

### Data Availability

All raw data and search results were uploaded onto the jPOST repository.^[^ ^51^ ^]^ The accession numbers are JPST001841 for JPOST and PXD036733 for ProteomeXchange.

## Conflict of Interest

The authors declare no conflict of interest.

## Supporting information

Supporting Information

## Data Availability

The data that support the findings of this study are available in the supplementary material of this article.

## References

[1] H. Sung , J. Ferlay , R. L. Siegel , M. Laversanne , I. Soerjomataram , A. Jemal , F. Bray , CA Cancer J Clin 2021, 71, 209. 33538338 10.3322/caac.21660

[2] T. Matsuoka , M. Yashiro , World J. Gastroenterol. 2018, 24, 2818. 30018477 10.3748/wjg.v24.i26.2818 PMC6048430

[3] E. Van Cutsem , X. Sagaert , B. Topal , K. Haustermans , H. Prenen , Lancet 2016, 388, 2654. 27156933 10.1016/S0140-6736(16)30354-3

[4] L. Necula , L. Matei , D. Dragu , A. I. Neagu , C. Mambet , S. Nedeianu , C. Bleotu , C. C. Diaconu , M. Chivu‐Economescu , World J. Gastroenterol. 2019, 25, 2029. 31114131 10.3748/wjg.v25.i17.2029 PMC6506585

[5] K. T. Schjoldager , Y. Narimatsu , H. J. Joshi , H. Clausen , Nat. Rev. Mol. Cell Biol. 2020, 21, 729. 33087899 10.1038/s41580-020-00294-x

[6] S. S. Pinho , C. A. Reis , Nat. Rev. Cancer 2015, 15, 540. 26289314 10.1038/nrc3982

[7] M. Wang , J. Zhu , D. M. Lubman , C. Gao , Clin. Chem. Lab. Med. 2019, 57, 407. 30138110 10.1515/cclm-2018-0379 PMC6785348

[8] R. J. Wong , A. Ahmed , R. G. Gish , Clin. Liver Dis. 2015, 19, 309. 25921665 10.1016/j.cld.2015.01.005

[9] H. Hanif , M. J. Ali , A. T. Susheela , I. W. Khan , M. A. Luna‐Cuadros , M. M. Khan , D. T.‐Y. Lau , World J. Gastroenterol. 2022, 28, 216. 35110946 10.3748/wjg.v28.i2.216 PMC8776528

[10] D. D. Engle , H. Tiriac , K. D. Rivera , A. Pommier , S. Whalen , T. E. Oni , B. Alagesan , E. J. Lee , M. A. Yao , M. S. Lucito , B. Spielman , B. Da Silva , C. Schoepfer , K. Wright , B. Creighton , L. Afinowicz , K. H. Yu , R. Grützmann , D. Aust , P. A. Gimotty , K. S. Pollard , R. H. Hruban , M. G. Goggins , C. Pilarsky , Y. Park , D. J. Pappin , M. A. Hollingsworth , D. A. Tuveson , Science 2019, 364, 1156. 31221853 10.1126/science.aaw3145 PMC6705393

[11] M. J. Kailemia , G. Xu , M. Wong , Q. Li , E. Goonatilleke , F. Leon , C. B. Lebrilla , Anal. Chem. 2018, 90, 208. 29049885 10.1021/acs.analchem.7b04202 PMC6200424

[12] L. Cao , T. M. Lih , Y. Hu , M. Schnaubelt , S.‐Y. Chen , Y. Zhou , C. Guo , M. Dong , W. Yang , R. V. Eguez , L. Chen , D. J. Clark , A. Sodhi , Q. K. Li , H. Zhang , Nat. Commun. 2022, 13, 3910. 35798744 10.1038/s41467-022-31472-4 PMC9262967

[13] J. Li , T. Zhao , J. Li , J. Shen , L. Jia , B. Zhu , L. Dang , C. Ma , D. Liu , F. Mu , L. Hu , S. Sun , Mol. Oncol. 2022, 16, 2135. 34855283 10.1002/1878-0261.13147 PMC9168967

[14] Z. Sun , B. Fu , G. Wang , L. Zhang , R. Xu , Y. Zhang , H. Lu , Natl. Sci. Rev. 2022, 10, nwac059. 36879659 10.1093/nsr/nwac059 PMC9985154

[15] T. Keser , M. Tijardovic , I. Gornik , E. Lukic , G. Lauc , O. Gornik , M. Novokmet , Mol. Cell. Proteomics 2021, 20, 100044. 33493676 10.1074/mcp.RA120.002433 PMC7950198

[16] J. Zhu , J. Huang , J. Zhang , Z. Chen , Y. Lin , G. Grigorean , L. Li , S. Liu , A. G. Singal , N. D. Parikh , D. M. Lubman , J. Proteome Res. 2020, 19, 3452. 32412768 10.1021/acs.jproteome.0c00270 PMC7429342

[17] L. Yang , Z. Sun , L. Zhang , Y. Cai , Y. Peng , T. Cao , Y. Zhang , H. Lu , Chem. Sci. 2019, 10, 9302. 32110292 10.1039/c9sc02491c PMC7006626

[18] M. Frantzi , A. Bhat , A. Latosinska , Clin. Transl. Med. 2014, 3, 7. 24679154 10.1186/2001-1326-3-7 PMC3994249

[19] S. Jeong , M. J. Oh , U. Kim , J. Lee , J.‐H. Kim , H. J. An , Expert Rev. Proteomics 2020, 17, 109. 32149536 10.1080/14789450.2020.1740091

[20] N. Bache , P. E. Geyer , D. B. Bekker‐Jensen , O. Hoerning , L. Falkenby , P. V. Treit , S. Doll , I. Paron , J. B. Müller , F. Meier , J. V. Olsen , O. Vorm , M. Mann , Mol. Cell. Proteomics 2018, 17, 2284. 30104208 10.1074/mcp.TIR118.000853 PMC6210218

[21] Y. Bian , R. Zheng , F. P. Bayer , C. Wong , Y.‐C. Chang , C. Meng , D. P. Zolg , M. Reinecke , J. Zecha , S. Wiechmann , S. Heinzlmeir , J. Scherr , B. Hemmer , M. Baynham , A.‐C. Gingras , O. Boychenko , B. Kuster , Nat. Commun. 2020, 11, 157. 31919466 10.1038/s41467-019-13973-x PMC6952431

[22] Y. Liu , R. Hüttenhain , B. Collins , R. Aebersold , Expert Rev. Mol. Diagn. 2013, 13, 811. 24138574 10.1586/14737159.2013.845089 PMC3833812

[23] L. Liu , B. Zhu , Z. Fang , N. Zhang , H. Qin , Z. Guo , X. Liang , Z. Yao , M. Ye , Anal. Chem. 2021, 93, 7473. 33973768 10.1021/acs.analchem.1c00645

[24] A. Kirwan , M. Utratna , M. E. O'dwyer , L. Joshi , M. Kilcoyne , Biomed Res. Int. 2015, 2015, 490531. 26509158 10.1155/2015/490531 PMC4609776

[25] P. E. Geyer , L. M. Holdt , D. Teupser , M. Mann , Mol Syst Biol 2017, 13, 942. 28951502 10.15252/msb.20156297 PMC5615924

[26] S. S. Pinho , S. Carvalho , R. Marcos‐Pinto , A. Magalhães , C. Oliveira , J. Gu , M. Dinis‐Ribeiro , F. Carneiro , R. Seruca , C. A. Reis , Trends Mol. Med. 2013, 19, 664. 23932995 10.1016/j.molmed.2013.07.003

[27] Z. Fang , H. Qin , J. Mao , Z. Wang , N. Zhang , Y. Wang , L. Liu , Y. Nie , M. Dong , M. Ye , Nat. Commun. 1900, 13, 1900. 10.1038/s41467-022-29530-y PMC8990002 35393418

[28] Y. Kizuka , N. Taniguchi , Biomolecules 2016, 6. 10.3390/biom6020025 PMC4919920 27136596

[29] H. Tian , E. Miyoshi , N. Kawaguchi , M. Shaker , Y. Ito , N. Taniguchi , M. Tsujimoto , N. Matsuura , Pathobiology 2008, 75, 288. 18931531 10.1159/000151709

[30] J. Fan , S. Wang , S. Yu , J. He , W. Zheng , J. Zhang , Glycoconj. J. 2012, 29, 323. 22736280 10.1007/s10719-012-9414-1

[31] K. Niimi , E. Yamamoto , S. Fujiwara , K. Shinjo , T. Kotani , T. Umezu , H. Kajiyama , K. Shibata , K. Ino , F. Kikkawa , Br. J. Cancer 1969, 107, 2012. 10.1038/bjc.2012.496 PMC3516685 23169300

[32] Y. Ide , E. Miyoshi , T. Nakagawa , J. Gu , M. Tanemura , T. Nishida , T. Ito , H. Yamamoto , Y. Kozutsumi , N. Taniguchi , Biochem. Biophys. Res. Commun. 2006, 341, 478. 16434023 10.1016/j.bbrc.2005.12.208

[33] J. A. Ferreira , A. Magalhães , J. Gomes , A. Peixoto , C. Gaiteiro , E. Fernandes , L. L. Santos , C. A. Reis , Cancer Lett. 2017, 387, 32. 26828132 10.1016/j.canlet.2016.01.044

[34] N. T. Marcos , A. Magalhães , B. Ferreira , M. J. Oliveira , A. S. Carvalho , N. Mendes , T. Gilmartin , S. R. Head , C. Figueiredo , L. David , F. Santos‐Silva , C. A. Reis , J. Clin. Invest. 2008, 118, 2325. 18483624 10.1172/JCI34324 PMC2381748

[35] (Eds.: V. Canzonieri , A. Giordano ), Gastric Cancer In The Precision Medicine Era: Diagnosis and Therapy, Springer International Publishing, Cham, 2019.

[36] Y. Bian , M. The , P. Giansanti , J. Mergner , R. Zheng , M. Wilhelm , A. Boychenko , B. Kuster , Anal. Chem. 2021, 93, 8687. 34124897 10.1021/acs.analchem.1c00738

[37] W. Yang , P. Shah , Y. Hu , S. Toghi Eshghi , S. Sun , Y. Liu , H. Zhang , Anal. Chem. 2017, 89, 11193. 29016103 10.1021/acs.analchem.7b03641 PMC5850954

[38] Q. Shu , M. Li , L. Shu , Z. An , J. Wang , H. Lv , M. Yang , T. Cai , T. Hu , Y. Fu , F. Yang , Mol. Cell. Proteomics 2020, 19, 672. 32102970 10.1074/mcp.RA119.001791 PMC7124471

[39] W. Zeng , S. Zheng , T. Su , J. Cheng , Y. Mao , Y. Zhong , Y. Liu , J. Chen , W. Zhao , T. Lin , F. Liu , G. Li , H. Yang , Y. Zhang , Front. Chem. 2022, 10, 839470. 35281567 10.3389/fchem.2022.839470 PMC8907888

[40] M. Xu , H. Jin , Z. Wu , Y. Han , J. Chen , C. Mao , P. Hao , X. Zhang , C.‐F. Liu , S. Yang , ACS Chem. Neurosci. 2022, 13, 1719. 35640092 10.1021/acschemneuro.2c00264

[41] Z. Wang , Z. Fang , L. Liu , H.e Zhu , Y. Wang , C. Zhao , Z. Guo , H. Qin , Y. Nie , X. Liang , M. Dong , M. Ye , Anal. Chem. 2023, 95, 7448. 37146305 10.1021/acs.analchem.2c04305

[42] S. Nie , A. Lo , J. Wu , J. Zhu , Z. Tan , D. M. Simeone , M. A. Anderson , K. A. Shedden , M. T. Ruffin , D. M. Lubman , J. Proteome Res. 2014, 13, 1873. 24571389 10.1021/pr400967x PMC3993962

[43] Y. Jin , J. Wang , X. Ye , Y. Su , G. Yu , Q. Yang , W. Liu , W. Yu , J. Cai , X. Chen , Y. Liang , Y. Chen , B. H. C. Wong , X. Fu , H. Sun , Br. J. Cancer 2016, 114, 532. 26908325 10.1038/bjc.2015.348 PMC4782198

[44] Y. Jin , Y. Yang , Y. Su , X. Ye , W. Liu , Q. Yang , J. Wang , X. Fu , Y. Gong , H. Sun , Glycoconj. J. 2019, 36, 57. 30607521 10.1007/s10719-018-09853-z

[45] S. Weiz , M. Wieczorek , C. Schwedler , M. Kaup , E. I. Braicu , J. Sehouli , R. Tauber , V. Blanchard , Electrophoresis 2016, 37, 1461. 26763099 10.1002/elps.201500518

[46] T. Caval , Y.‐H. Lin , M. Varkila , K. R. Reiding , M. J. M. Bonten , O. L. Cremer , V. Franc , A. J. R. Heck , Front. Immunol. 2020, 11, 608466. 33519818 10.3389/fimmu.2020.608466 PMC7840657

[47] Y. Jin , W. Wang , Q. Wang , Y. Zhang , K. R. Zahid , U. Raza , Y. Gong , Cancer Cell Int. 2022, 22, 156. 35439996 10.1186/s12935-022-02572-4 PMC9019971

[48] S. Tyanova , T. Temu , P. Sinitcyn , A. Carlson , M. Y. Hein , T. Geiger , M. Mann , J. Cox , Nat. Methods 2016, 13, 731. 27348712 10.1038/nmeth.3901

[49] X. Robin , N. Turck , A. Hainard , N. Tiberti , F. Lisacek , J.‐C. Sanchez , M. Müller , BMC Bioinf. 2011, 12, 77. 10.1186/1471-2105-12-77 PMC3068975 21414208

[50] T. Metsalu , J. Vilo , Nucleic Acids Res. 2015, 43, W566. 25969447 10.1093/nar/gkv468 PMC4489295

[51] S. Okuda , Y. Watanabe , Y. Moriya , S. Kawano , T. Yamamoto , M. Matsumoto , T. Takami , D. Kobayashi , N. Araki , A. C. Yoshizawa , T. Tabata , N. Sugiyama , S. Goto , Y. Ishihama , Nucleic Acids Res. 2017, 45, D1107. 27899654 10.1093/nar/gkw1080 PMC5210561

