# Supplementary material for: Robust Glycoproteomics Platform Reveals a Tetra‐Antennary Site‐Specific Glycan Capping with Sialyl‐Lewis Antigen for Early Detection of Gastric Cancer
Source: Adv Sci (Weinh). 2023 Dec 12;11(9):2306955. doi: 10.1002/advs.202306955 (PMC10916543; doi:10.1002/advs.202306955)
Supplement: Supplementary file 1 — Supporting Information [file ADVS-11-2306955-s001.pdf]

ADVANCED SCIENCE
Open Access

Supporting Information

for *Adv. Sci.*, DOI 10.1002/advs.202306955

Robust Glycoproteomics Platform Reveals a Tetra-Antennary Site-Specific Glycan Capping with Sialyl-Lewis Antigen for Early Detection of Gastric Cancer

Luyao Liu, Lei Liu, Yan Wang, Zheng Fang, Yangyang Bian, Wenyao Zhang, Zhongyu Wang, Xianchun Gao, Changrui Zhao, Miaomiao Tian, Xiaoyan Liu, Hongqiang Qin, Zhimou Guo, Xinmiao Liang, Mingming Dong, Yongzhan Nie* and Mingliang Ye**

Supporting Information

Robust glycoproteomics platform reveals a tetra-antennary site-specific glycan capping with sialyl-Lewis antigen for early detection of gastric cancer

Luyao Liu, Lei Liu, Yan Wang, Zheng Fang, Yangyang Bian, Wenyao Zhang, Zhongyu Wang, Xianchun Gao, Changrui Zhao, Miaomiao Tian, Xiaoyan Liu, Hongqiang Qin, Zhimou Guo, Xinmiao Liang, Mingming Dong, Yongzhan Nie*, Mingliang Ye**

Luyao Liu, Lei Liu, and Yan Wang contributed equally in this work.

Luyao Liu[1,6], Lei Liu[1,6], Yan Wang[1], Zheng Fang[1], Zhongyu Wang[1], Xiaoyan Liu[1], Hongqiang Qin[1], Zhimou Guo[1], Xinmiao Liang[1], and Mingliang Ye[1,3,6*]
Address[1]: CAS Key Laboratory of Separation Science for Analytical Chemistry, Dalian Institute of Chemical Physics, Chinese Academy of Sciences, Dalian, 116023, China
Address[3]: State Key Laboratory of Medical Proteomics, Beijing, 102206, China
Address[6]: University of Chinese Academy of Sciences, Beijing, 101408, China
E-mail: mingliang@dicp.ac.cn

Changrui Zhao[2] and Mingming Dong[2,*]
Address: MOE Key Laboratory of Bio-Intelligent Manufacturing, School of Bioengineering, Dalian University of Technology, Dalian, 116024, China
E-mail: dongmm@dlut.edu.cn

Yangyang Bian[4]
Address[4]: The College of Life Sciences, Northwest University, Xi'an, 710127, China

Wenyao Zhang[5], Xianchun Gao[5], Miaomiao Tian[5], and Yongzhan Nie[5*]
Address: State Key Laboratory of Cancer Biology, Xijing Hospital of Digestive Diseases, Fourth Military Medical University, Xi'an, 710068, China
E-mail: yongznie@fmmu.edu.cn

Table of content:

1. Supplementary methods.
2. Figure S1-S16.
3. Table S1-S4.
4. Table S5 (excel).

Supplementary methods

Serum N-glycopeptides analyzed by nanoflow LC-MS/MS. Nanoflow LC-MS/MS analysis was performed on the same LC and MS instruments as microflow LC-MS/MS. Samples were first loaded onto the capillary trap column (C18-AQ, 1.9 μm, 3 cm × 200 μm inner diameter, Agela technologies) and separated by the capillary analysis column (C18-AQ, 1.9 μm, 20 cm × 150 μm inner diameter, Ammerbuch) at a flow rate of 600 nL/min. The overall 60 min LC gradient was described as follows: held at 4% B to load peptides for 4 min, from 4% to 45% B for 46 min, from 45% to 95% B for 2 min, and held on 95% B to clean the system for 4 min, and finally back to 4% B to equilibrate system for 4 min. MS analysis was operated in the data-dependent mode to switch between MS and MS/MS acquisition. Full scan MS spectra were collected from 350 to 1,800 m/z at a resolution of 70,000, with an AGC target of 1e6 and IT of 20 ms. MS/MS scans were obtained at a resolution of 35,000 using an isolation window of 2 m/z, with an AGC target of 2e5 and maximum IT of 100 ms. Glycopeptide fragmentation was performed by stepped HCD with normalized energy of 20%, 30% and 40%.

N-glycopeptide characterization using pGlyco 3.0 software. Raw files of plasma N-glycopeptides were analyzed by pGlyco 3.0 software[1] for comparison with Glyco-Decipher identification results. GPSMs with a total FDR < 1% were reserved as qualified identifications. Other search parameters adopted in pGlyco 3.0 were the same as those used in Glyco-Decipher.

Identification algorithm of Glyco-Decipher. Glyco-Decipher [2] was applied to identify glycopeptides in this study. Its algorithm includes the following steps:

1) In silico deglycosylation and peptide identificaiton. Taking the advantage of common core structure of N-glycans (i.e., Man(3)GlcNAc(2)), Glyco-Decipher first matches Y-ions of core structure by finding fragment ions in mass spectra with specific mass gap of monosaccharides. For example, the mass gap between Y2 and Y1 ion is 203 Da for GlcNAc in N-glycan core structure. After matching core structure Y ions, mass value of peptide backbone of the glycopeptide is precisely derived from the m/z value of Y1 ion, which is usually high abundant in glycopeptide spectra. Then an in silico de-glycosylated peptide spectrum is generated in Glyco-Decipher by removing all oxonium ions and Y ions and replacing the precursor by derived peptide mass. And the deglyco-spectrum is identified by proteomics database searching to determine the peptide sequence in glycopeptide. And only PSM results with FDR<0.01 are retained or further analysis.

2) Spectrum Expansion. The fragmentation pattern of the same peptide has been proved to be highly similar even when it are modified with different glycans [2] . To improve the identification sensitivity, the fragmentation pattern of peptide backbones is extracted and utilized to match peptides in glycopeptide spectra that remain unidentified in the first step,

and the strategy is named as "Spectrum Expansion". The obtained average peptide fragmentation pattern in the first step is used in spectrum expansion scoring:

$$\mathrm{Score_{PSM}} = \mathrm{Score_{Peptide}} + \mathrm{Score_{Core}} = \mathrm{coefficient} \times \sum_{i=1}^{n}\left(\frac{\mathrm{Intensity}_i}{\mathrm{frequency}_i}\right)^{\alpha} + \sum_{j=1}^{m}\left(\frac{\mathrm{Intensity}_j}{\mathrm{frequency}_j}\right) \quad (1)$$

where intensity is the relative intensity of the matched peptide fragment ions; frequency is the number of fragment ions generated by other peptides in the mass tolerance window; and coefficient is the cosine similarity between the fragmentation pattern in the PSM and the average peptide pattern (this value should be near 1.0 if two patterns match well or zero if a total random match occurs). α is set to be 0.3 in Glyco-Decipher; n is the number of matched fragment ions of peptide backbone, and m is the number of matched fragment ions of N-glycan core structure. The score to assess a peptide-spectrum match consists of two parts: the score of peptide ions, which is to assess the peptide identification with the matched peptide ions and their intensity pattern; the score of core structure ions, which aims to avoid identification of peptides with incorrect mass, especially to avoid the incorrect assignment of peptides with shared peptide fragment ions (e.g. peptides with different number of missed cleavages).

For quality control in spectrum expansion, decoy spectra (m/z value of each fragment ion in MS2 is shifted with 1-30 m/z randomly) and linear tail-fit method[3] is used to calculate the e-value, i.e. the expectation value that the obtained match is valid, of each PSM in spectrum expansion. Then all PSMs, including target and decoy PSMs, are sorted in decreasing order of spectral e-values. And the FDR for PSMs with e-value < threshold t is calculated with the formula of FDR = (2*Ndecoy)/(Ndecoy + Ntarget), where N is the number of PSMs with e-value < t. Instead of setting fixed score threshold for all peptides, the score threshold is calculated dynamically based on the e-value method during spectrum expansion for each peptide. Then PSMs with FDR<0.01 and with at least 3 matched core structure fragment ions (in which the Y1 (peptide+HexNAc) ion is required) are retained for glycan part identification.

3) Glycan Annotation. After the identification of peptide part, the mass value of glycan part is derived from the corrected precursor mass. The glycan mass is matched to a build-in glycan database, which is downloaded from GlyTouCan website (https://glytoucan.org/). The theoretical fragment ions of candidate database glycan are enumerated based on the structure information (encoded in WURCS 2.0 format) and are matched to the glycopeptide spectrum to evaluate the confidence of glycan part identification result. The function below is used in Glyco-Decipher to rank glycan candidates based on matched fragment ions between theoretical and experimental values:

$$\mathrm{Score_{Glycan}} = \sum_{i=1}^{n}\left(\frac{\mathrm{Intensity}_i}{\mathrm{frequency}_i}\right)^{\alpha} \quad (2)$$

where intensity is the relative intensity of the matched fragment ions and frequency is the number of glycan ions generated by other glycopeptides in the mass tolerance window. And

the peptide and top-ranked glycan identifications of Glyco-Decipher are reported as the final result. For more detailed algorithm description of Glyco-Decipher, please refer to our recent publication[2].

Figure S1

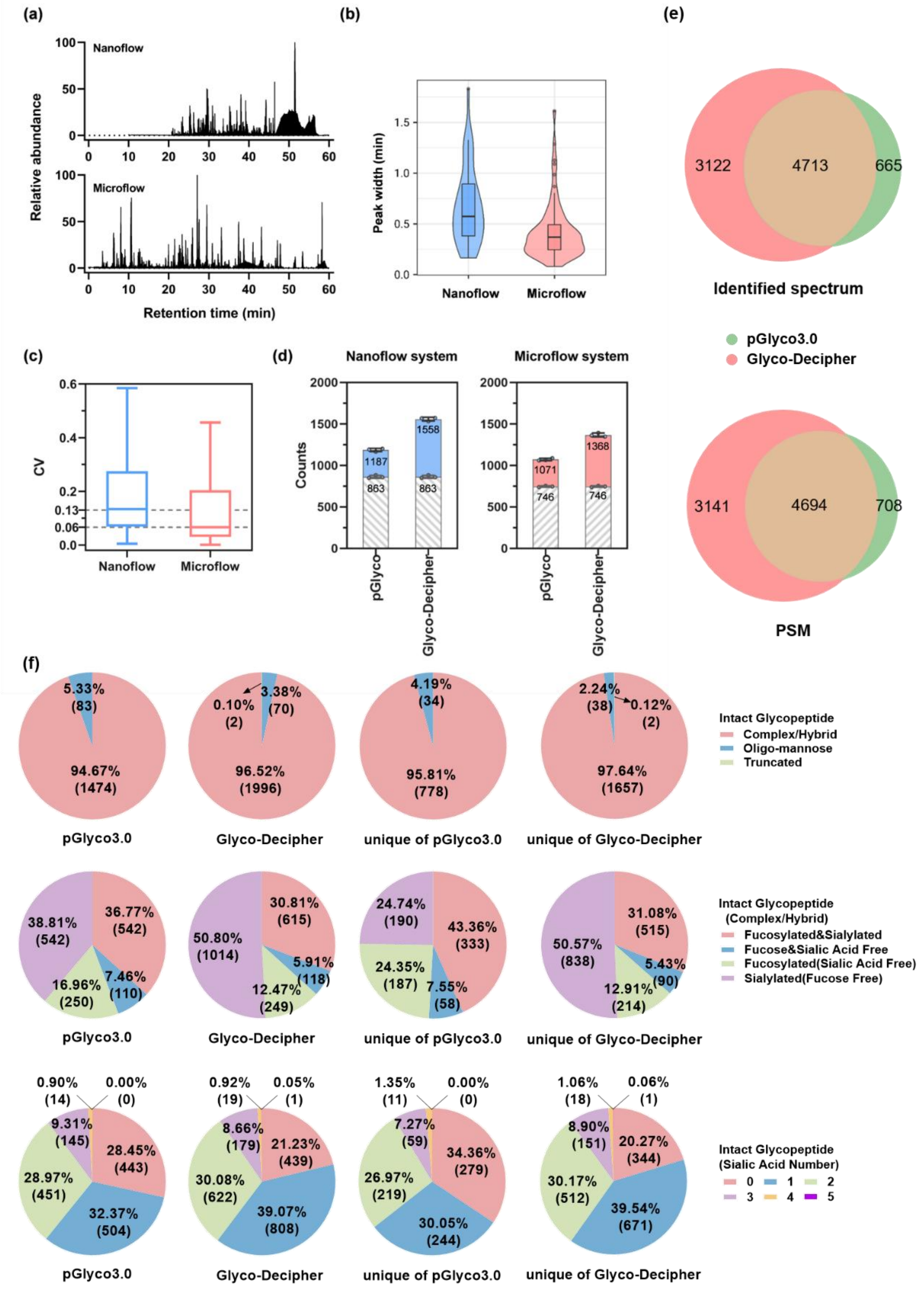
(a)
Nanoflow
Microflow
Relative abundance
Retention time (min)
(b)
Peak width (min)
Nanoflow
Microflow
(c)
CV
Nanoflow
Microflow
(d)
Nanoflow system
Microflow system
Counts
pGlyco
Glyco-Decipher
(e)
3122
4713
665
Identified spectrum
pGlyco3.0
Glyco-Decipher
3141
4694
708
PSM
(f)
Intact Glycopeptide
Complex/Hybrid
Oligo-mannose
Truncated
pGlyco3.0
Glyco-Decipher
unique of pGlyco3.0
unique of Glyco-Decipher
Intact Glycopeptide (Complex/Hybrid)
Fucosylated&Sialylated
Fucose&Sialic Acid Free
Fucosylated(Sialic Acid Free)
Sialylated(Fucose Free)
Intact Glycopeptide (Sialic Acid Number)

(continued)

(g)

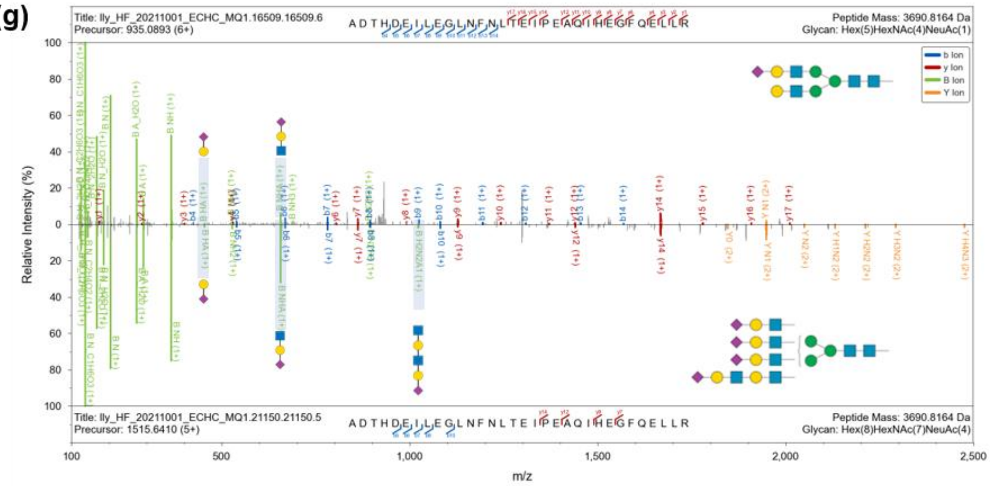

(h)

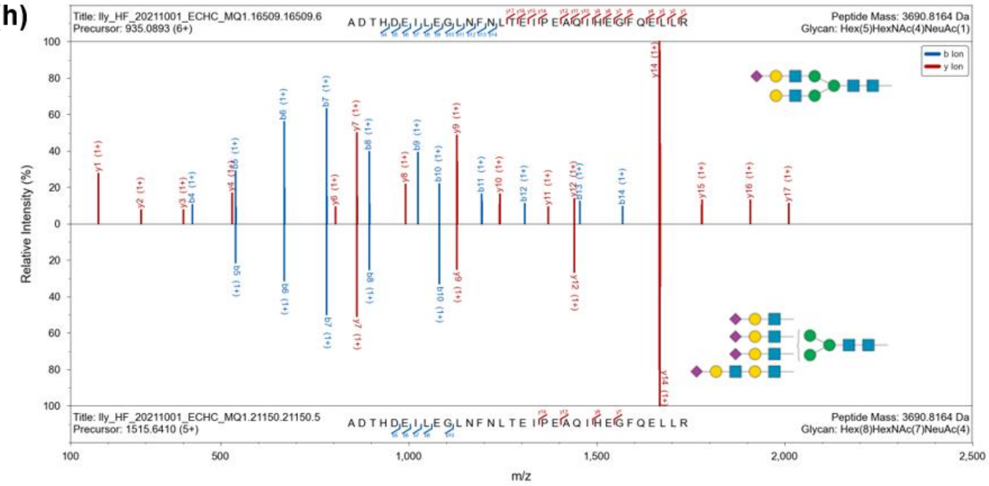

Figure S1. Performance evaluation of N-glycopeptide identification and quantification in microflow and nanoflow LC-MS/MS analysis. Glycopeptides enriched by the automated method from pooled plasma samples were analyzed using microflow or nanoflow LC coupled with the same MS instrument, repsectively. Considering the capacity of column loadings, the nanoflow system used 1 μL of starting pooled plasma, while the microflow system used 5 μL. (a) Base peak chromatograms of glycopeptides using a 60-min gradient in the nanoflow (above) and microflow (below) LC-MS/MS, respectively. (b) Violin plots of chromatographic peak width distributions. (c) Coefficient of variation (CV) of quantified glycopeptides, of which quantitation were measured by Glyco-Decipher software. (d) The identification number of intact N-glycopeptides searched by pGlyco 3.0 and Glyco-Decipher, respectively. Hatched sections showed the overlap between the results of two software. (e) Venn diagrams of identified spectra and PSM from the MS data acquired by microflow LC-MS/MS system. For plasma N-glycoproteomics analysis in triplicate, Glyco-Decipher and pGlyco 3.0 have idetified a total of 7835 and 5378 glycopeptide spectra, respectively. Glyco-Decipher covers 87.63% (4713/5378) of spectra of pGlyco 3.0, where 99.6 % (4694/4713) of PSMs were the same, indicating that both Glyco-Decipher and pGlyco3.0 have high confidence in identifying glycopeptide spectra. (f) Glycoform distribution in the total GPSMs and unique part of GPSMs. (g) Example of intact glycopeptides with additional spectrum identification through

spectrum expansion in Glyco-Decipher. Glycopeptide spectrum matched by in silico deglycosylation (top) and spectrum expansion method (bottom). (h) Normalized peptide fragment ions of intact glycopeptides shown in (g).

The glycoform distribution of intact glycopeptides identified by Glyco-Decipher was quite similar to that of pGlyco 3.0 except a relative higher percentage of sialylated glycopeptides (Figure S1f). Serum protein N-glycosylation is generally highly sialylated, however, the fragmentation of glycopeptides bearing negatively charged sialic acids in HCD are inefficient and the obtained MS/MS spectra often lack sufficient fragment ions for peptide sequence identification by pGlyco 3.0. For example, the bottom spectra in Figure S1g&h from the glycopeptide with 4 terminal sialic acids cannot be identified by pGlyco 3.0 due to the lack of sufficient peptide fragments. But the peptide sequence could be successfully identified by Glyco-Decipher with spectrum expansion strategy. In Glyco-Decipher, the spectra of the glycopeptide with one terminal sialic acid (the top spectra in Figure S1g&h) could easily yield peptide sequence identification by in silico deglycosylation because of the rich peptide fragments. The peptide fragmentation pattern of the identified spectrum was then exploited to identify the peptide sequences of the spectra with poor peptide fragments (the bottom spectra in Figure S1g&h). The spectrum expansion strategy of Glyco-Decipher improves the identification sensitivity of glycopeptide spectra with poor peptide fragmentation by exploiting the high similarity in peptide fragmentation patterns of glycopeptides with shared peptide sequences. Thus, Glyco-Decipher has an advantage in identification of multi-sialylated glycopeptides for revealing the micro-heterogeneity of serum protein N-glycosylation.

Figure S2

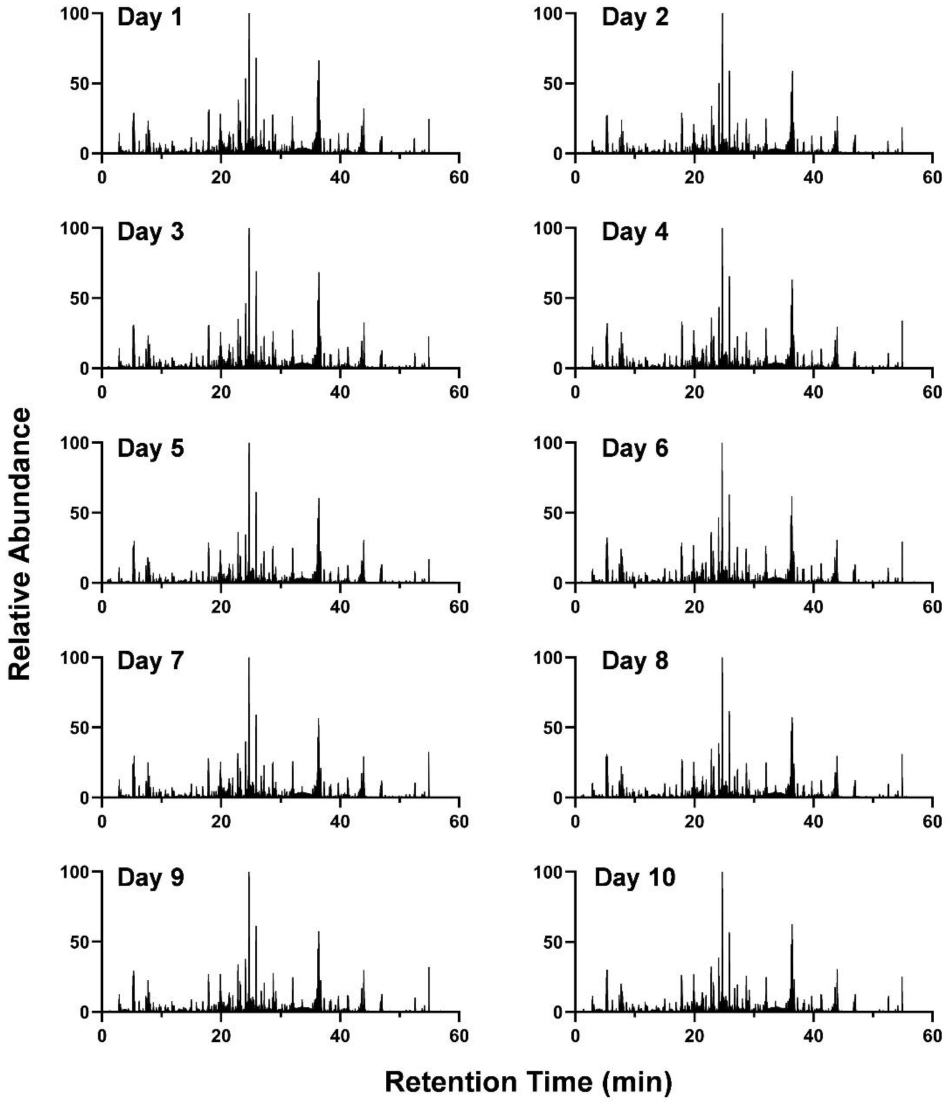

Figure S2. Base peak chromatograms of ten MS-QC glycopeptide samples. One MS-QC per day for a total of ten days.

Figure S3

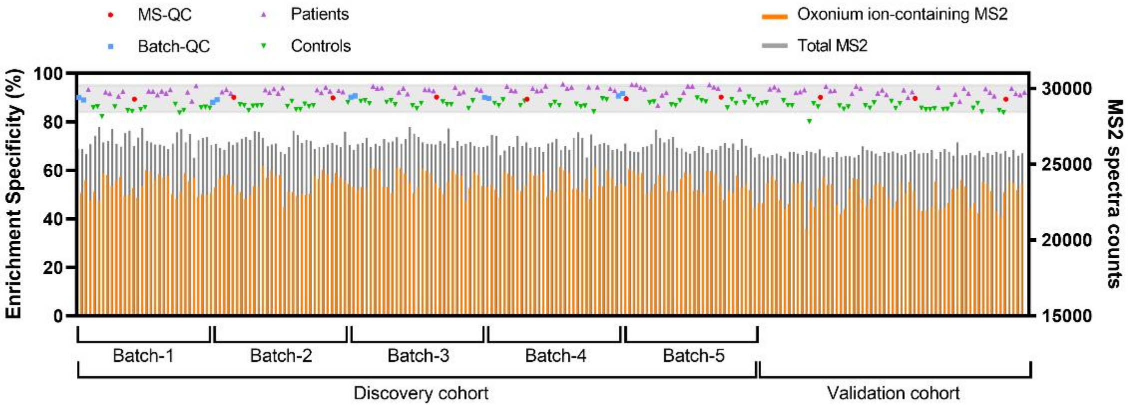

Figure S3. Enrichment specificity evaluation of the HRN platform. Enrichment specificity (dot plot) and MS2 spectra counts (bar plot) of ten MS-QCs, ten batch-QCs, and all 200 individuals in discovery and validation cohorts. Enrichment specificity was assessed by the ratio of the number of the oxonium ion-containing spectra to the total MS2 spectra detected by Glyco-Decipher. The sample order in the x-axis corresponds to the injection order of LC-MS/MS analysis.

Figure S4

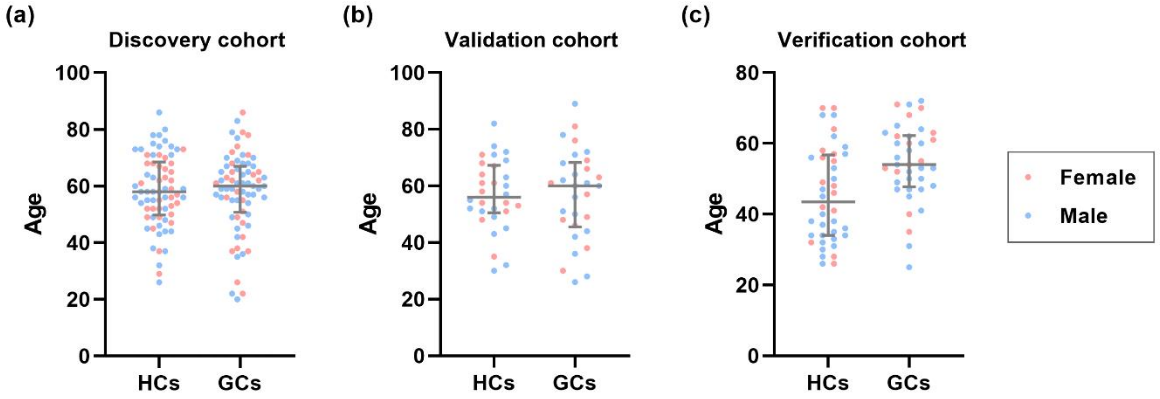

Figure S4. Age and gender distributions of HCs and GCs in three cohorts. (a) Discovery cohort; (b) Validation cohort; (c) Verification cohort. HCs, healthy controls; GCs, GC patients. Error bar, median with interquartile range.

Figure S5

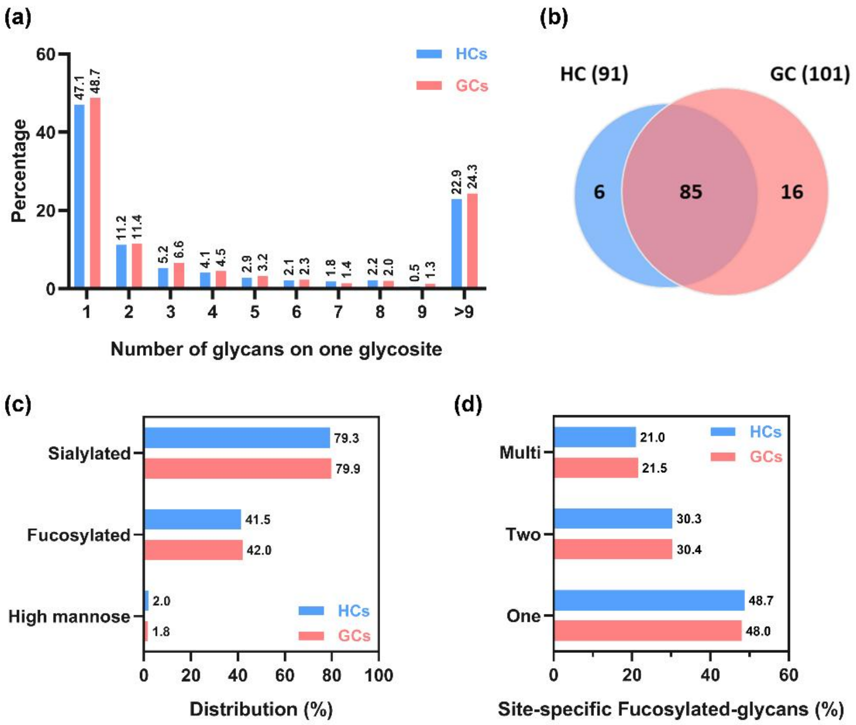

Figure S5. Site-specific glycan distributions in the discovery cohort. (a) Distribution of different numbers of glycans on glycosites. (b) Overlap of glycoproteins containing ten or more N-glycans at one glycosites between GCs and HCs. (c) Distribution of sialylated glycans, fucosylated glycans, and high mannose. (d) Distribution of fucosylated glycans with one, two, or multi fucoses.

Figure S6

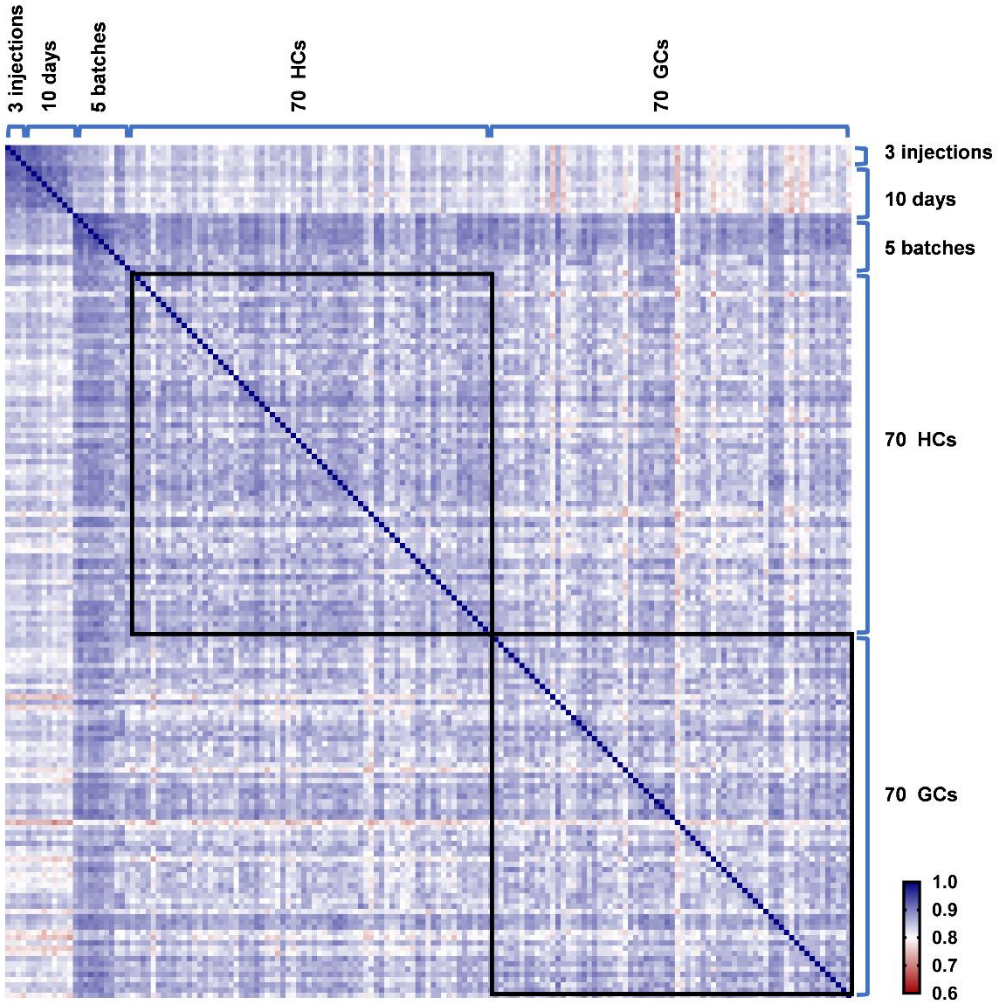

Figure S6. Heatmap for Pearson correlation coefficients of quantified N-glycopeptides. The overall coefficients of three injections, ten MS-QCs and ten batch-QCs were higher than that of 140 clinical samples, revealing personal differences.

Figure S7

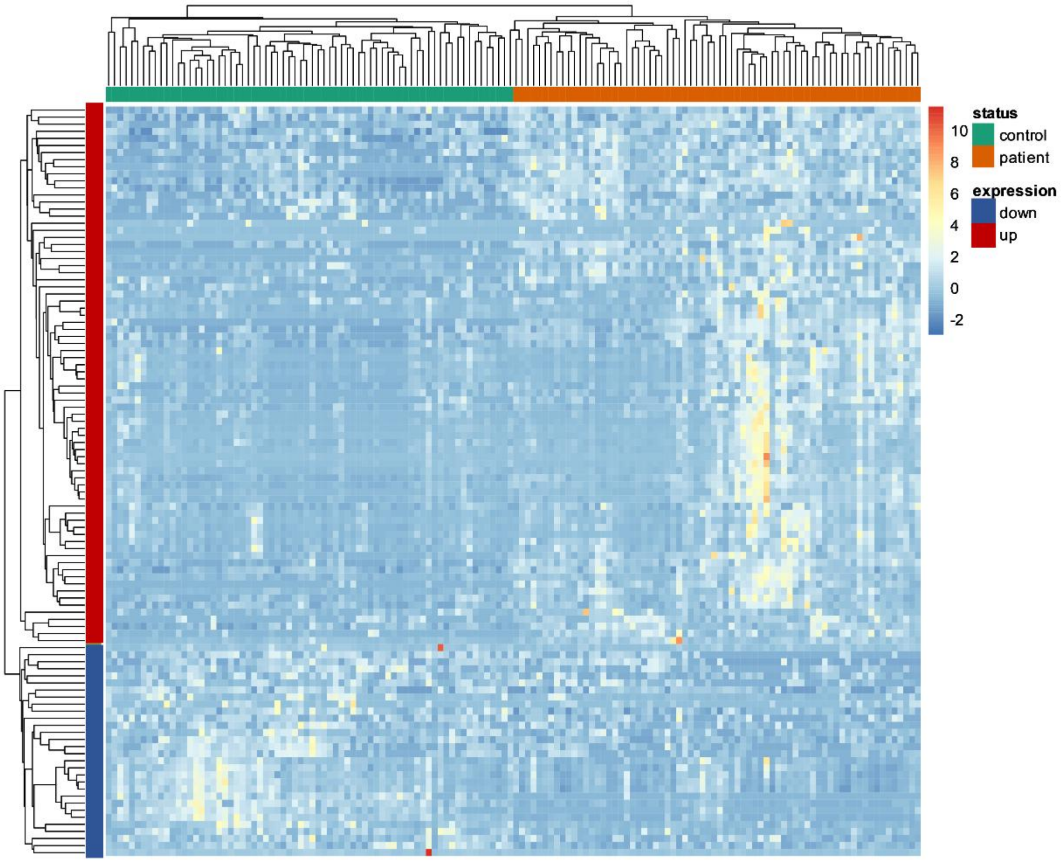

Figure S7. Clustering analysis of 106 dysregulated glycopeptides in discovery cohort. Dysregulated glycopeptides could form two distinct clusters in GCs and HCs.

Figure S8

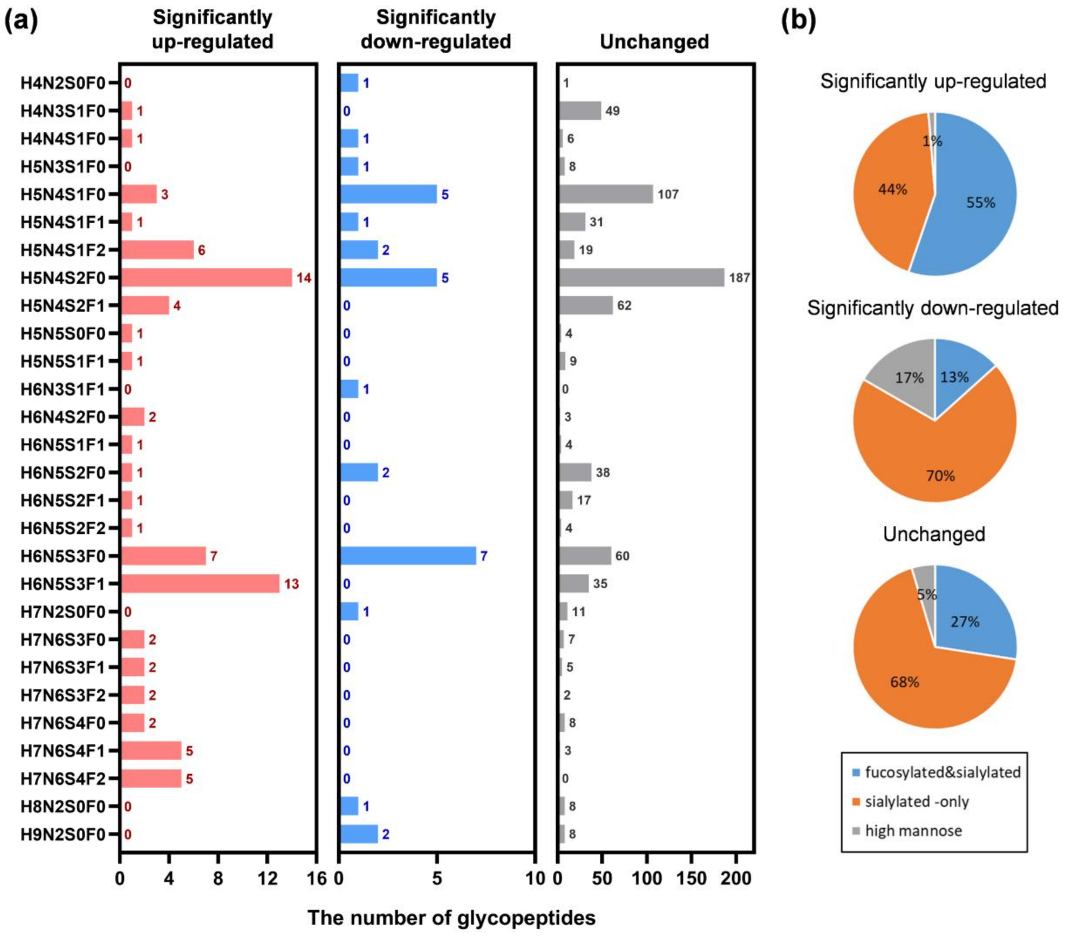

Figure S8. Glycopeptides with significantly up-regulation, down-regulation, or unchanged expression in GC in the discovery cohort. (a) Comparison of the number glycopeptides with the same glycan compositions. (b) Distribution of glycopeptides modified with both fucosylated and sialylated, sialylated-only, and high mannose.

Figure S9

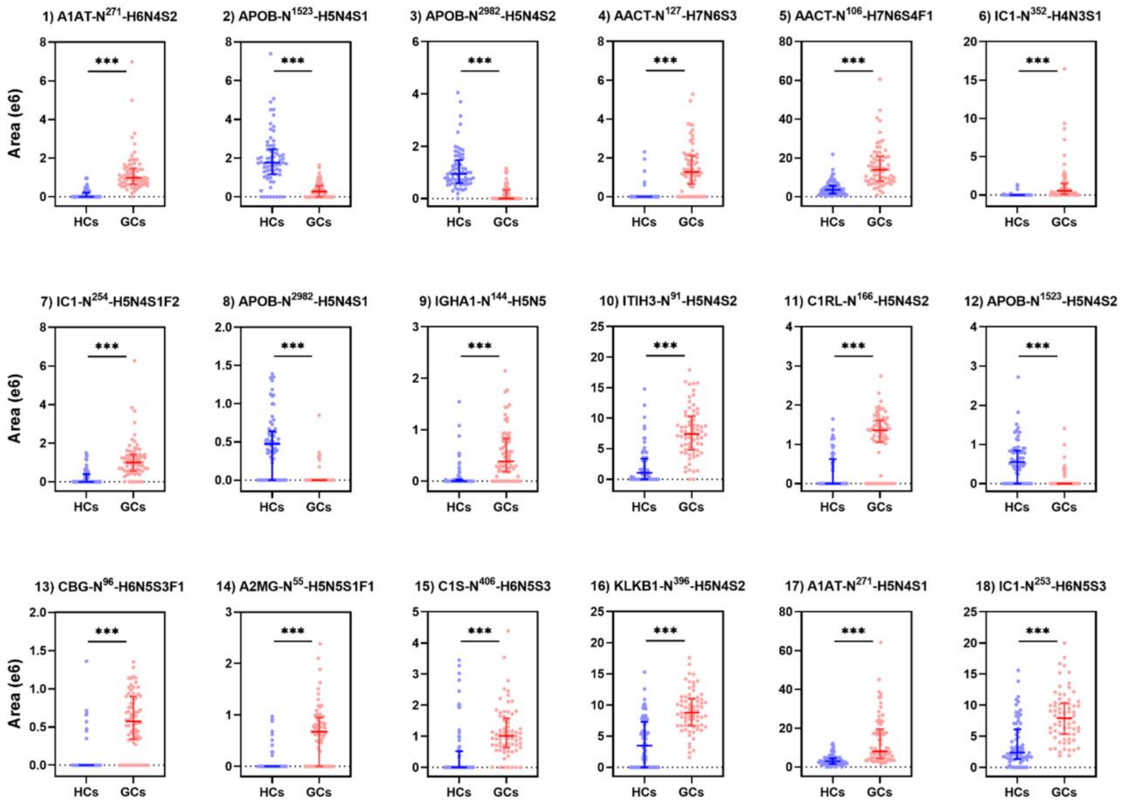

Figure S9. Quantitation distributions of top 18 signatures in discovery cohort. ***, p value < 0.001.

Figure S10

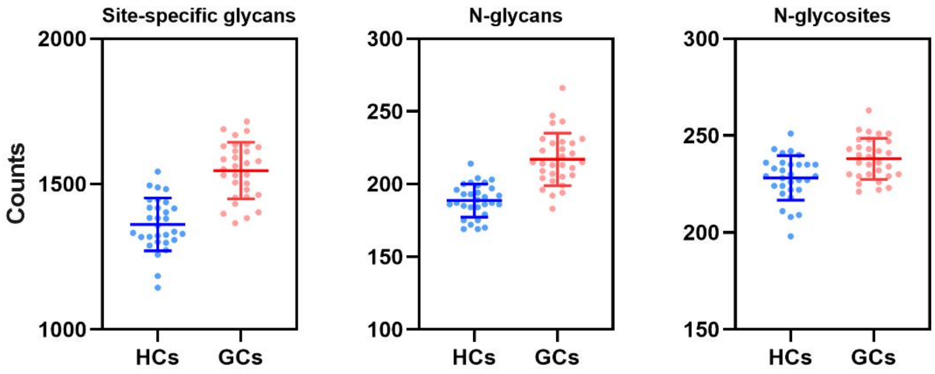

Figure S10. The number of site-specific glycans, N-glycans, and N-glycosites identified in validation cohort. These identifications of validation cohort showed similar trends to that of discovery cohort.

Figure S11

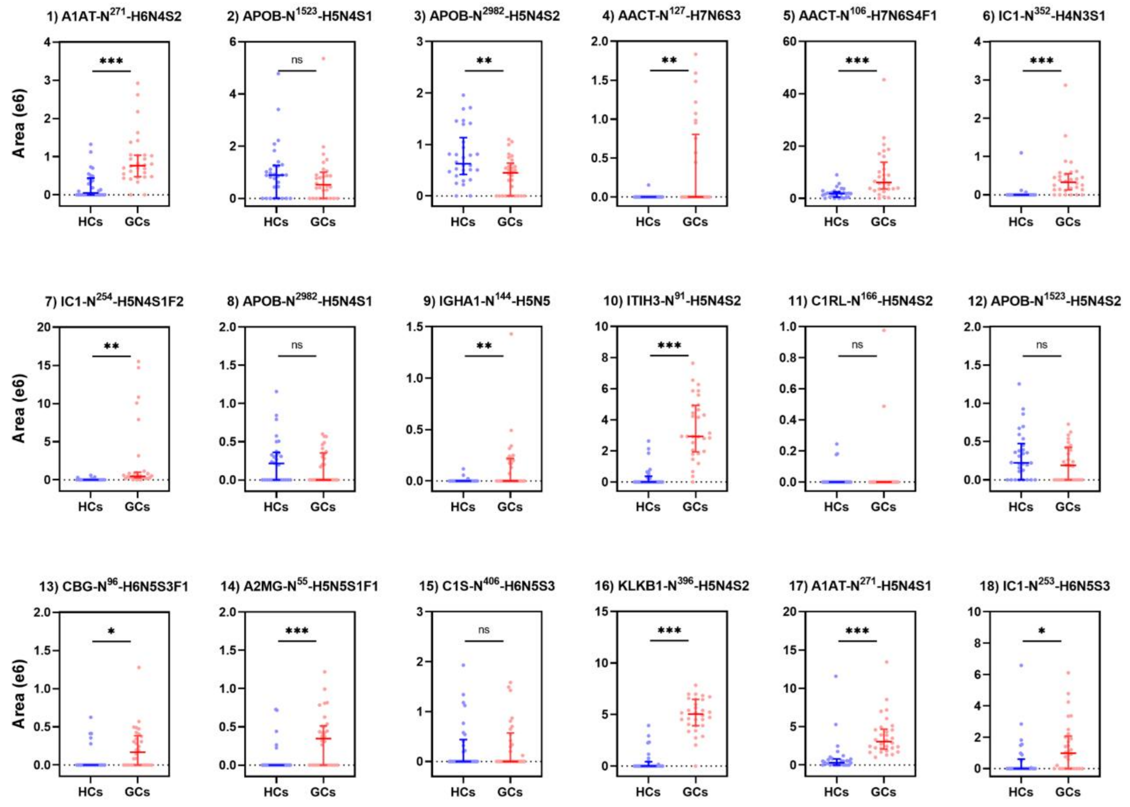

Figure S11. Quantitation distributions of top 18 signatures in validation cohort. ***, p value < 0.001; **, p value < 0.01; *, p value < 0.05; ns, p value > 0.05.

Figure S12

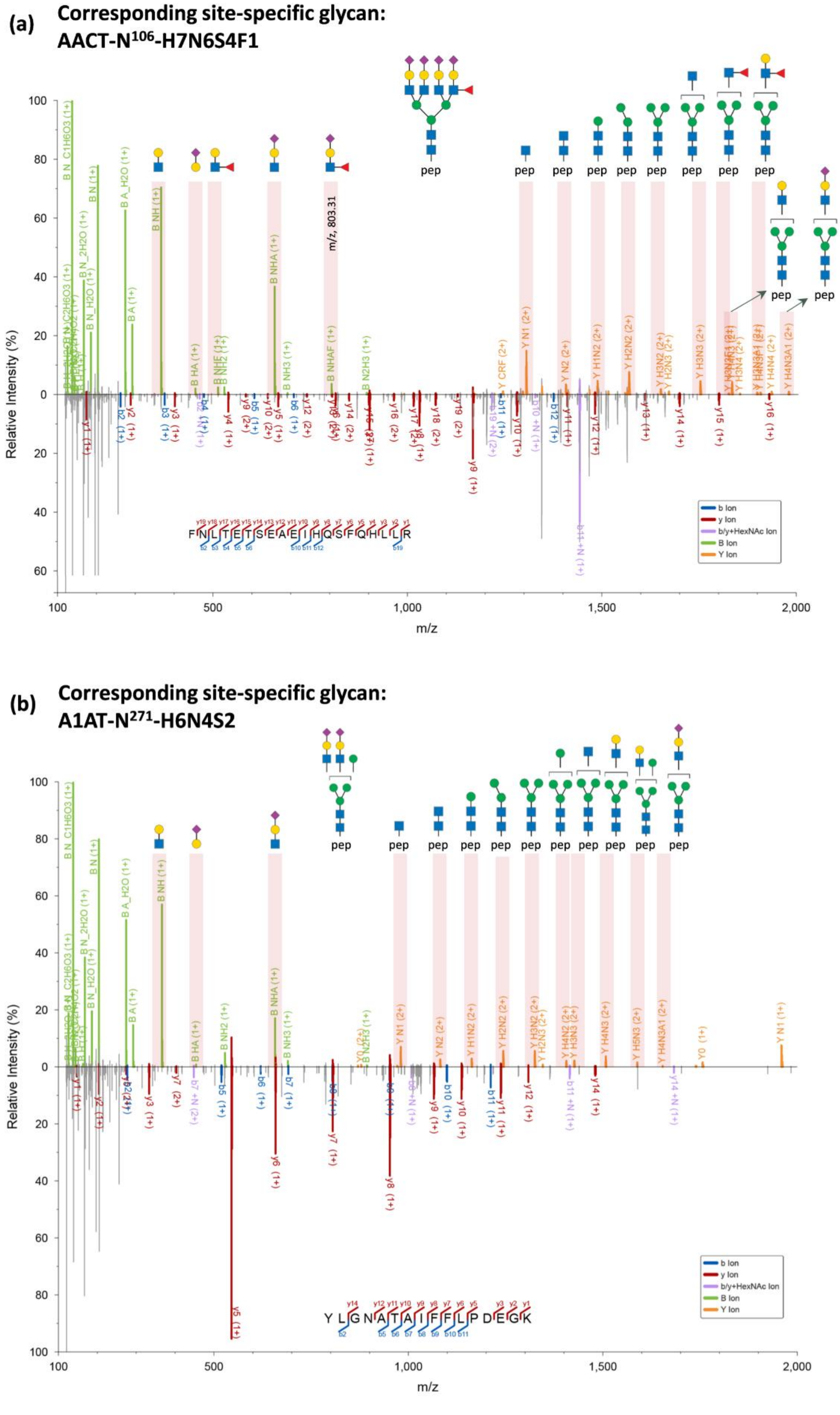
(a)
Corresponding site-specific glycan:
AACT-N106-H7N6S4F1
Relative Intensity (%)
m/z
m/z, 803.31
FNLTETSEAEIHQSFQHLLR
b Ion
y Ion
b/y+HexNAc Ion
B Ion
Y Ion
(b)
Corresponding site-specific glycan:
A1AT-N271-H6N4S2
Relative Intensity (%)
m/z
YLGNATAIFFLPDEGK
b Ion
y Ion
b/y+HexNAc Ion
B Ion
Y Ion

(continued)

(c) **Corresponding site-specific glycan: IC1-N[352]-H4N3S1**

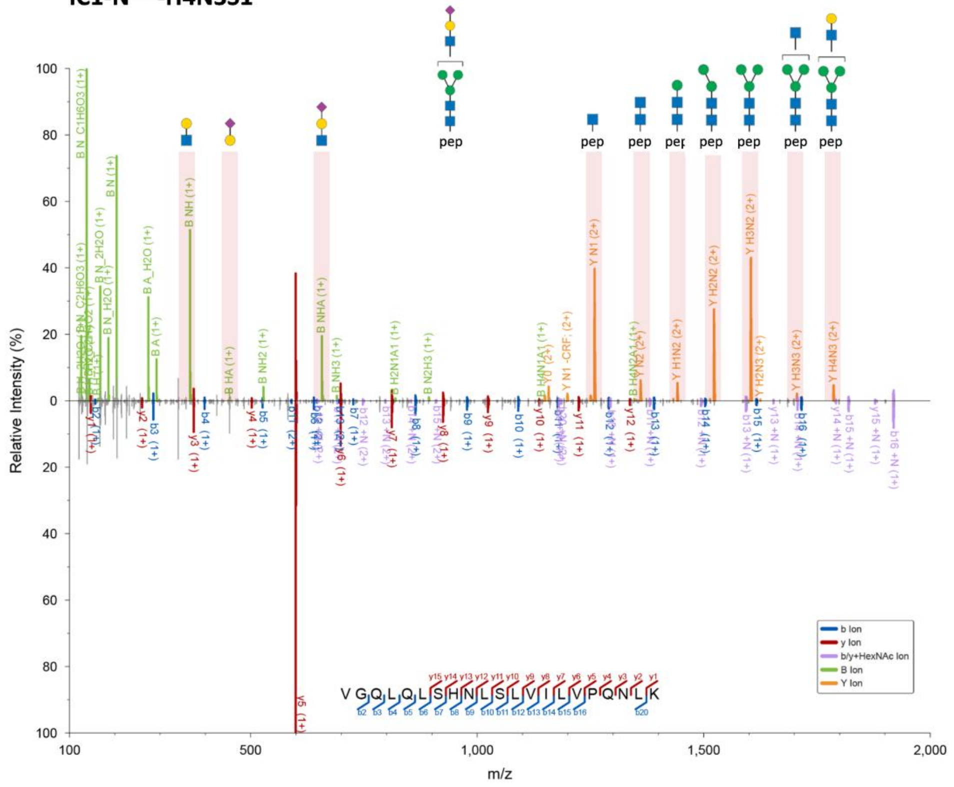

(d) **Corresponding site-specific glycan: ITIH3-N[91]-H5N4S2**

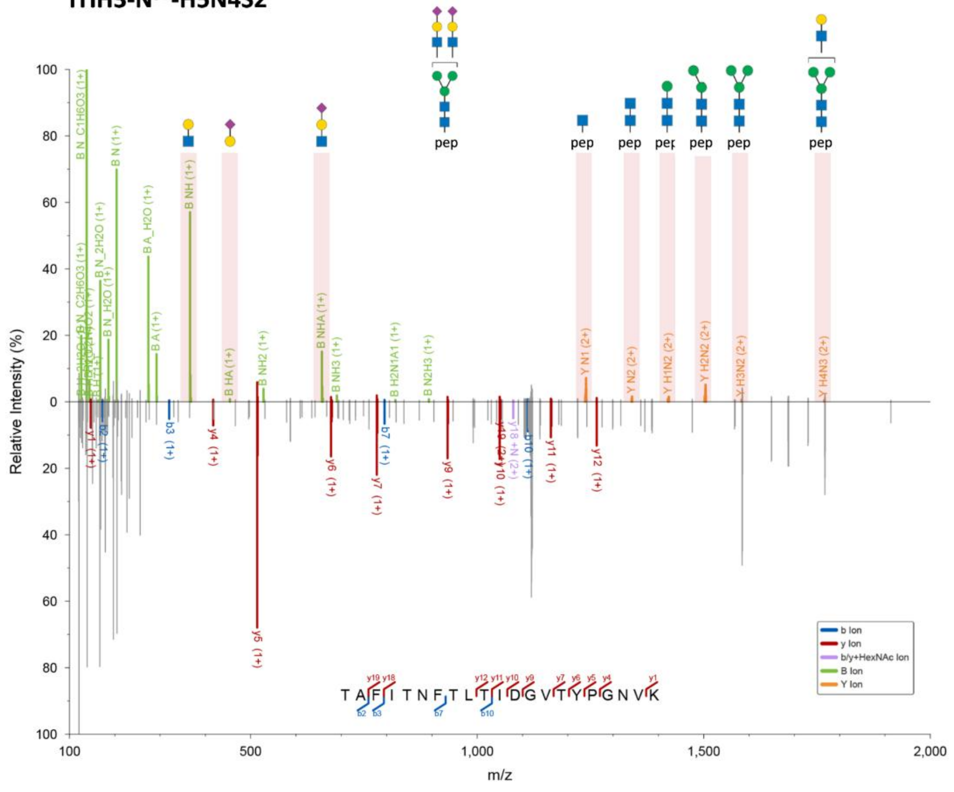

(continued)

Figure S12. Representative MS2 spectra of glycopeptides corresponding to (a) AACT-N^{106}-H7N6S4F1, (b) A1AT-N^{271}-H6N4S2, (c) IC1-N^{352}-H4N3S1, and (d) ITIH3-N^{91}- H5N4S2. Paired spectrum with B/Y fragment ions (top) and b/y fragment ions (bottom). Figure S12a is the same as Figure 4a.

Figure S13

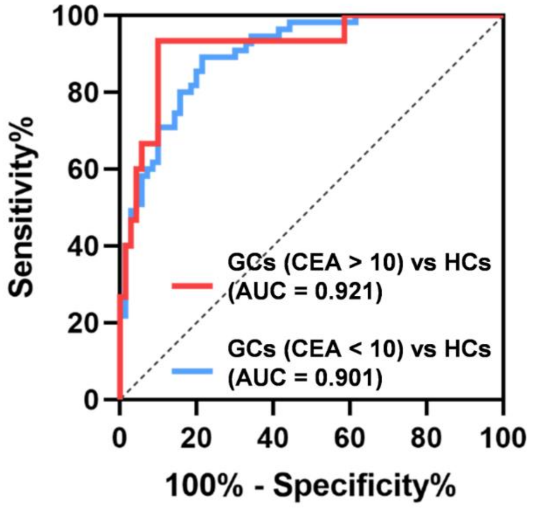

Figure S13. ROC curves for diagnosing GCs with CEA > 10 ng/mL (red line) or GCs with CEA < 10 ng/mL (blue line) using AACT-N106-H7N6S4F1 in the discovery cohort.

Figure S14

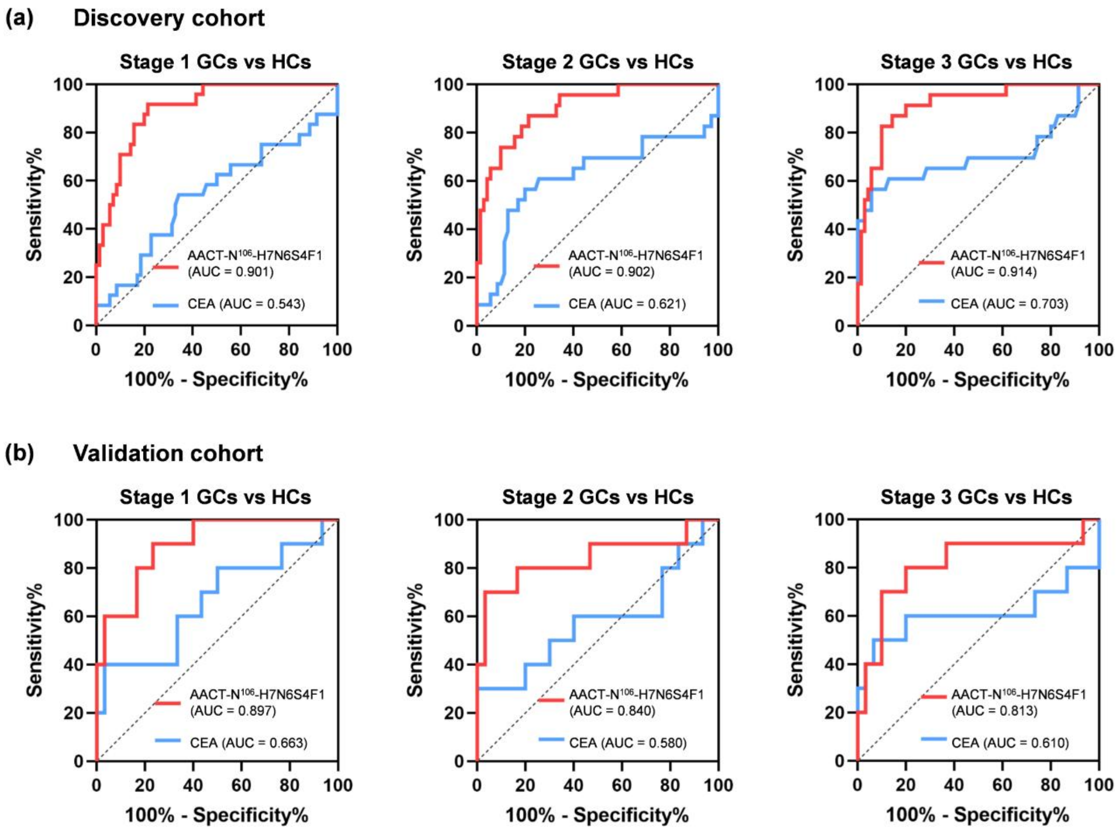

Figure S14. ROC curves for diagnosing three stages of GCs using serum CEA and AACT-N^{106}-H7N6S4F1. (a) The discovery cohort. (b) The validation cohort.

Figure S15

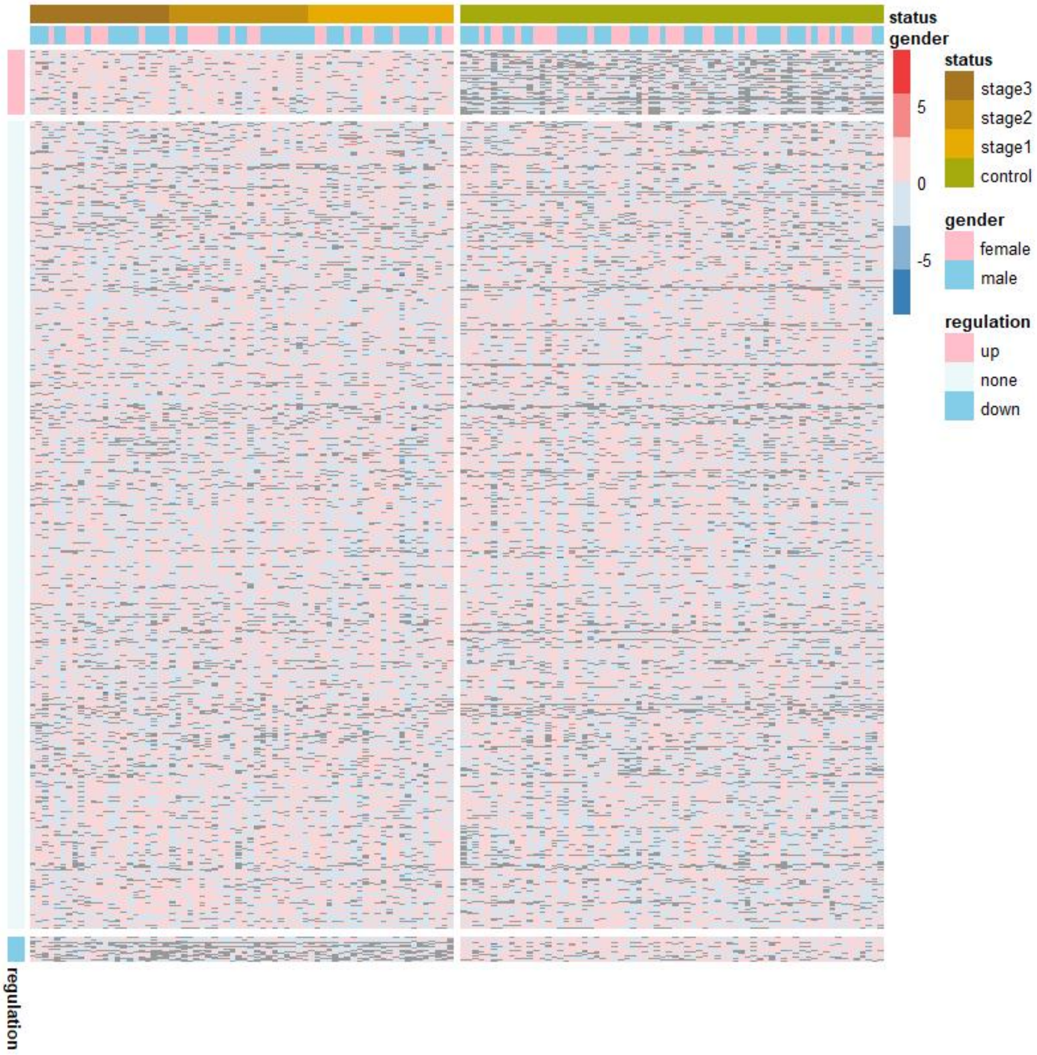

Figure S15. Heatmap of quantitative values of 1,053 N-glycopeptides in the discovery cohort. 76 glycopeptides were significantly upregulated, 30 were significantly downregulated, and 947 were unchanged (GCs vs HCs). Missing value in the heatmap were shown in gray.

Figure S16

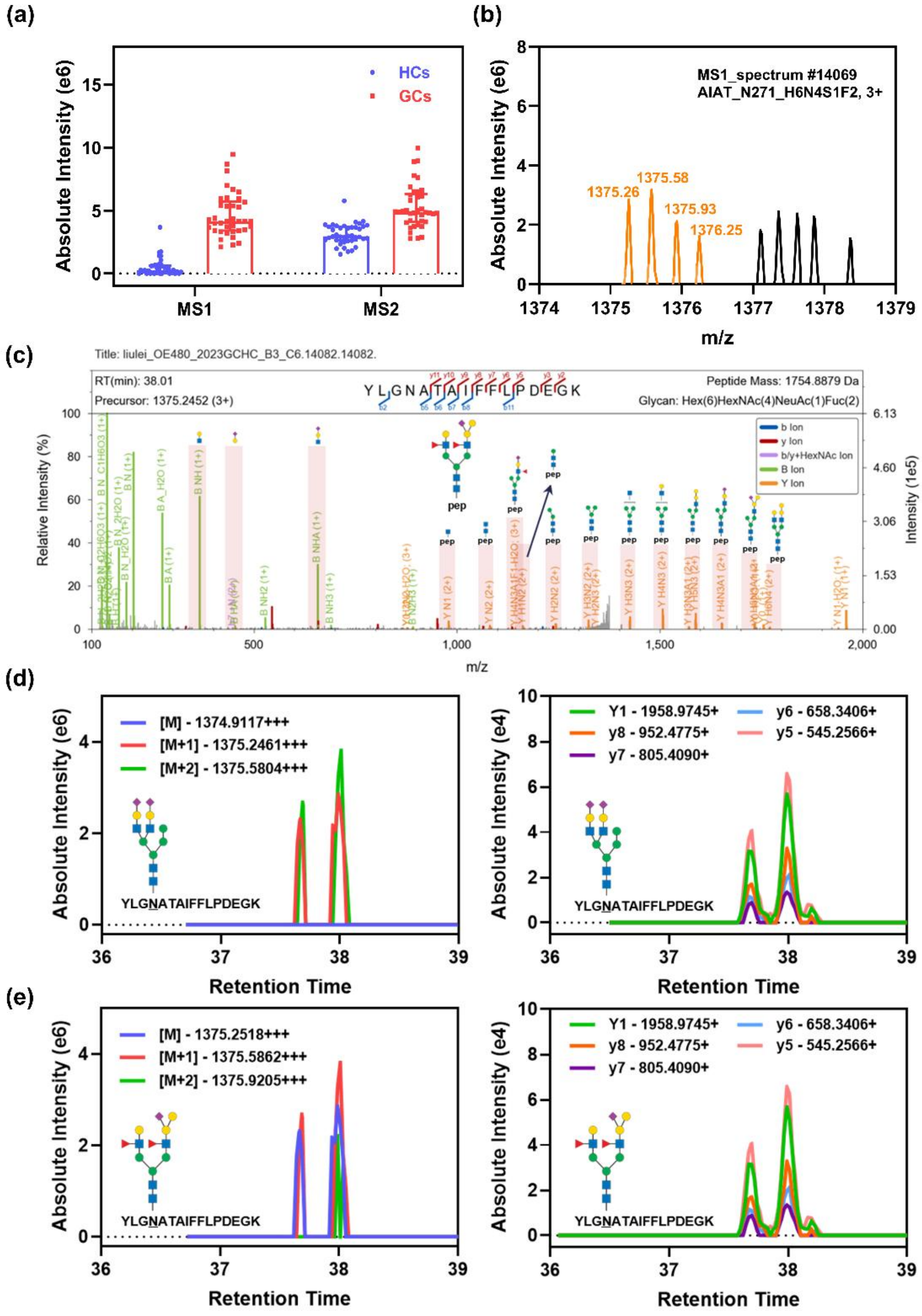

Figure S16. Comparison of MS1-based quantification by Glyco-Decipher and MS2-based quantification by Skyline. (a) Abundance distributions of A1AT-N^{271}-H6N4S2 using MS1-based and MS2-based quantification approaches, respectively. GCs had similar quantitave values in both approaches,while HCs had much higher quantitative values in MS2-based approach than in MS1-based approach. We then checked all MS2 spectra used in Skyline and the corresponding MS1 spectra. (b) One typical MS1 spectrum belonged to A1AT-N^{271}-H6N4S1F2 (3+) based on MS2 spectrum (#14082) idetification. And this MS1

spectrum did not contain the monoisotopic peak at m/z 1374.9 for A1AT-N^{271}-H6N4S2 (3+). (c) The MS2 spectrum (#14082) mentioned above through careful spectra deciphering indicated the correct recognition. Glycopeptides for A1AT-N^{271}-H6N4S2 and A1AT-N^{271}-H6N4S1F2 had similar glycans attached to the same peptide backbone. Skyline had difficulty distinguishing these glycopeptides since they had similar high abundant Y and b/y ions for quantification. We further compared the extracted ion chromatograms of glycopeptides for A1AT-N^{271}-H6N4S2 (d) and A1AT-N^{271}-H6N4S1F2 (e) recognized by Skyline. Their fragment ion extraction profiles (right panels) for Y1 and y ions were almost identical. Nevertheless, the corresponding full-scan MS ion extraction profiles (left panels) showed that monoisotopic peak for A1AT-N^{271}-H6N4S2 was missing, indicating the wrong extraction; while A1AT-N^{271}-H6N4S1F2 performed accurate signal. Therefore, the accuracy of MS2-based glycopeptide quantification by Skyline is currently hard to determine.

Table S1. Top 18 important signatures in a random forest machine learning model. Each important signature here represents one intact N-glycopeptide and corresponds to a distinct site-specific glycan.

Rank	Intact N-glycopeptide	Corresponding site-specific glycan	MeanDecrease Gini value
1	Hex(6)HexNAc(4)NeuAc(2)@YLGNATAIFFLPDEGK	A1AT-N^{271}-H6N4S2F0	6.441
2	Hex(5)HexNAc(4)NeuAc(1)@FNSSYLQGTNQITGR	APOB-N^{1523}-H5N4S1F0	4.213
3	Hex(5)HexNAc(4)NeuAc(2)@VNQNLVYESGSLNFSK	APOB-N^{2982}-H5N4S2F0	4.177
4	Hex(7)HexNAc(6)NeuAc(3)@TLNQSSDELQLSMGNAMFVK	AACT-N^{127}-H7N6S3F0	4.085
5	Hex(7)HexNAc(6)NeuAc(4)Fuc(1)@GLKFNLTETSEAEIHQSFQHLLR	AACT-N^{106}-H7N6S4F1	3.939
6	Hex(4)HexNAc(3)NeuAc(1)@VGQLQLSHNLSLVILVPQNLK	IC1-N^{352}-H4N3S1F0	3.528
7	Hex(5)HexNAc(4)NeuAc(1)Fuc(2)@VLSNNSDANLELINTWVAK	IC1-N^{254}-H5N4S1F2	2.649
8	Hex(5)HexNAc(4)NeuAc(1)@VNQNLVYESGSLNFSK	APOB-N^{2982}-H5N4S1F0	2.309
9	Hex(5)HexNAc(5)@PALEDLLLGSEANLTCTLTGLR 16,car;	IGHA1-N^{144}-H5N5S0F0	2.192
10	Hex(5)HexNAc(4)NeuAc(2)@TAFITNFTLTIDGVTYPGNVK	ITIH3-N^{91}-H5N4S2F0	2.157
11	Hex(5)HexNAc(4)NeuAc(2)@GFLALYQTVAVNYSQPISEASR	C1RL-N^{166}-H5N4S2F0	2.008
12	Hex(5)HexNAc(4)NeuAc(2)@FNSSYLQGTNQITGR	APOB-N^{1523}-H5N4S2F0	1.820
13	Hex(6)HexNAc(5)NeuAc(3)Fuc(1)@AQLLQGLGFNLTER	CBG-N^{96}-H6N5S3F1	1.702
14	Hex(5)HexNAc(5)NeuAc(1)Fuc(1)@GCVLLSYLNETVTVSASLESVR 2,car;	A2MG-N^{55}-H5N5S1F1	1.605
15	Hex(6)HexNAc(5)NeuAc(3)@YTCEEPYYYMENGGGGEYHCAGNGSWVNEVLGPELPK 3,car;20,car;	C1S-N^{406}-H6N5S3F0	1.536
16	Hex(5)HexNAc(4)NeuAc(2)@IVGGTNSSWGEWPWQVSLQVK	KLKB1-N^{396}-H5N4S2F0	1.304
17	Hex(5)HexNAc(4)NeuAc(1)@YLGNATAIFFLPDEGKLQHLENELTHDIITK	A1AT-N^{271}-H5N4S1F0	1.188
18	Hex(6)HexNAc(5)NeuAc(3)@VLSNNSDANLELINTWVAK	IC1-N^{253}-H6N5S3F0	1.027

Table S2. AUC values of 18 important signatures in the discovery and validation cohorts. Each signature here represents one intact N-glycopeptide and corresponds to a distinct site-specific glycan.

Rank	Corresponding site-specific glycan	AUC（discovery cohort）	AUC（validation cohort）
1	A1AT-N^{271}-H6N4S2	0.957	0.850
2	APOB-N^{1523}-H5N4S1	0.883	0.593
3	APOB-N^{2982}-H5N4S2	0.921	0.699
4	AACT-N^{127}-H7N6S3	0.871	0.344
5	AACT-N^{106}-H7N6S4F1	0.901	0.846
6	IC1-N^{352}-H4N3S1	0.863	0.864
7	IC1-N^{254}-H5N4S1F2	0.861	0.909
8	APOB-N^{2982}-H5N4S1	0.836	0.556
9	IGHA1-N^{144}-H5N5	0.819	0.294
10	ITIH3-N^{91}-H5N4S2	0.873	0.947
11	C1RL-N^{166}-H5N4S2	0.834	0.513
12	APOB-N^{1523}-H5N4S2	0.820	0.588
13	CBG-N^{96}-H6N5S3F1	0.842	0.703
14	A2MG-N^{55}-H5N5S1F1	0.825	0.729
15	C1S-N^{406}-H6N5S3	0.792	0.467
16	KLKB1-N^{396}-H5N4S2	0.833	0.965
17	A1AT-N^{271}-H5N4S1	0.847	0.917
18	IC1-N^{253}-H6N5S3	0.812	0.699

Table S3. List of precursor ions of targeted glycopeptides used in PRM acquisition mode.

Site-specific glycan	Peptide sequence	Precursor m/z	Charge (z)	Start time (min)	End time (min)
A1AT-N[271]-H6N4S2	YLGNATAIFFLPDEGK	1374.9185	3+	35.21	40.21
	YLGNATAIFFLPDEGK	1031.4421	4+	35.00	40.00
	YLGNATAIFFLPDEGKLQHLENELTHDIITK	1182.3451	5+	39.32	44.32
AACT-N[106]-H7N6S4F1	FNLTETSEAEIHQSFQHLLR	1516.6371	4+	33.68	38.68
	FNLTETSEAEIHQSFQHLLR	1213.5095	5+	33.67	38.67
	GLKFNLTETSEAEIHQSFQHLLR	1273.1499	5+	32.32	37.32
	GLKFNLTETSEAEIHQSFQHLLR	1061.1249	6+	32.34	37.34
IC1-N[352]-H4N3S1	VGQLQLSHNLSLVILVPQNLK	1287.9734	3+	33.50	38.50
	VGQLQLSHNLSLVILVPQNLK	966.2407	4+	33.49	38.49
ITIH3-N[91]-H5N4S2	TAFITNFTLTIDGVTYPGNVK	1493.0017	3+	38.13	43.13
	TAFITNFTLTIDGVTYPGNVK	1120.0029	4+	38.14	43.14

Table S4. List of fragment ions of targeted glycopeptide for MS2-based quantification used in Skyline.

Site-specific glycan	Fragment ions for quantification
A1AT-N271-H6N4S2F0	y5 - 545.2566+
	Y1 - 1958.9745+
	y8 - 952.4775+
	y6 - 658.3406+
	y7 - 805.4090+
AACT-N106-H7N6S4F1	Y1 - 1302.1404++
	Y4 - 1565.7329++
	Y3 - 1484.7065++
	Y2 - 1403.6801++
	Y0 - 1200.6007++
IC1-N352-H4N3S1F0	Y1 - 1258.7259++
	Y5 - 1603.3448++
	Y4 - 1522.3184++
	Y0 - 1157.1862++
	Y3 - 1441.2920++
ITIH3-N91-H5N4S2F0	y5 - 514.2984+
	y10 - 1049.5262+
	y7 - 778.4094+
	y6 - 677.3617+
	y9 - 934.4993+

References

[1] W.-F. Zeng, W.-Q. Cao, M.-Q. Liu, S.-M. He, P.-Y. Yang, *Nat Methods* 2021, 18, 1515.
[2] Z. Fang, H. Qin, J. Mao, Z. Wang, N. Zhang, Y. Wang, L. Liu, Y. Nie, M. Dong, M. Ye, *Nat Commun* 2022, 13, 1900.
[3] D. Fenyö and R. C. Beavis, *Anal Chem* 2003, 75, 768.
